# Synthetic Thiol and Selenol Derived Amino Acids for Expanding the Scope of Chemical Protein Synthesis

**DOI:** 10.3389/fchem.2021.826764

**Published:** 2022-02-14

**Authors:** Ivy Guan, Kayla Williams, Joanna Shu Ting Liu, Xuyu Liu

**Affiliations:** ^1^ School of Chemistry, Faculty of Science, The University of Sydney, Sydney, NSW, Australia; ^2^ The Heart Research Institute, The University of Sydney, Sydney, NSW, Australia; ^3^ School of Medical Sciences, Faculty of Medicine and Health, The University of Sydney, Sydney, NSW, Australia

**Keywords:** peptide ligation, unnatural amino acid, cysteine, chemical protein synthesis, semisynthesis, protein modification, desulfurization, deselenization

## Abstract

Cells employ post-translational modifications (PTMs) as key mechanisms to expand proteome diversity beyond the inherent limitations of a concise genome. The ability to incorporate post-translationally modified amino acids into protein targets via chemical ligation of peptide fragments has enabled the access to homogeneous proteins bearing discrete PTM patterns and empowered functional elucidation of individual modification sites. Native chemical ligation (NCL) represents a powerful and robust means for convergent assembly of two homogeneous, unprotected peptides bearing an N-terminal cysteine residue and a C-terminal thioester, respectively. The subsequent discovery that protein cysteine residues can be chemoselectively desulfurized to alanine has ignited tremendous interest in preparing unnatural thiol-derived variants of proteogenic amino acids for chemical protein synthesis following the ligation-desulfurization logic. Recently, the 21st amino acid selenocysteine, together with other selenyl derivatives of amino acids, have been shown to facilitate ultrafast ligation with peptidyl selenoesters, while the advancement in deselenization chemistry has provided reliable bio-orthogonality to PTMs and other amino acids. The combination of these ligation techniques and desulfurization/deselenization chemistries has led to streamlined synthesis of multiple structurally-complex, post-translationally modified proteins. In this review, we aim to summarize the latest chemical synthesis of thiolated and selenylated amino-acid building blocks and exemplify their important roles in conquering challenging protein targets with distinct PTM patterns.

## Introduction

**SCHEME 1:**
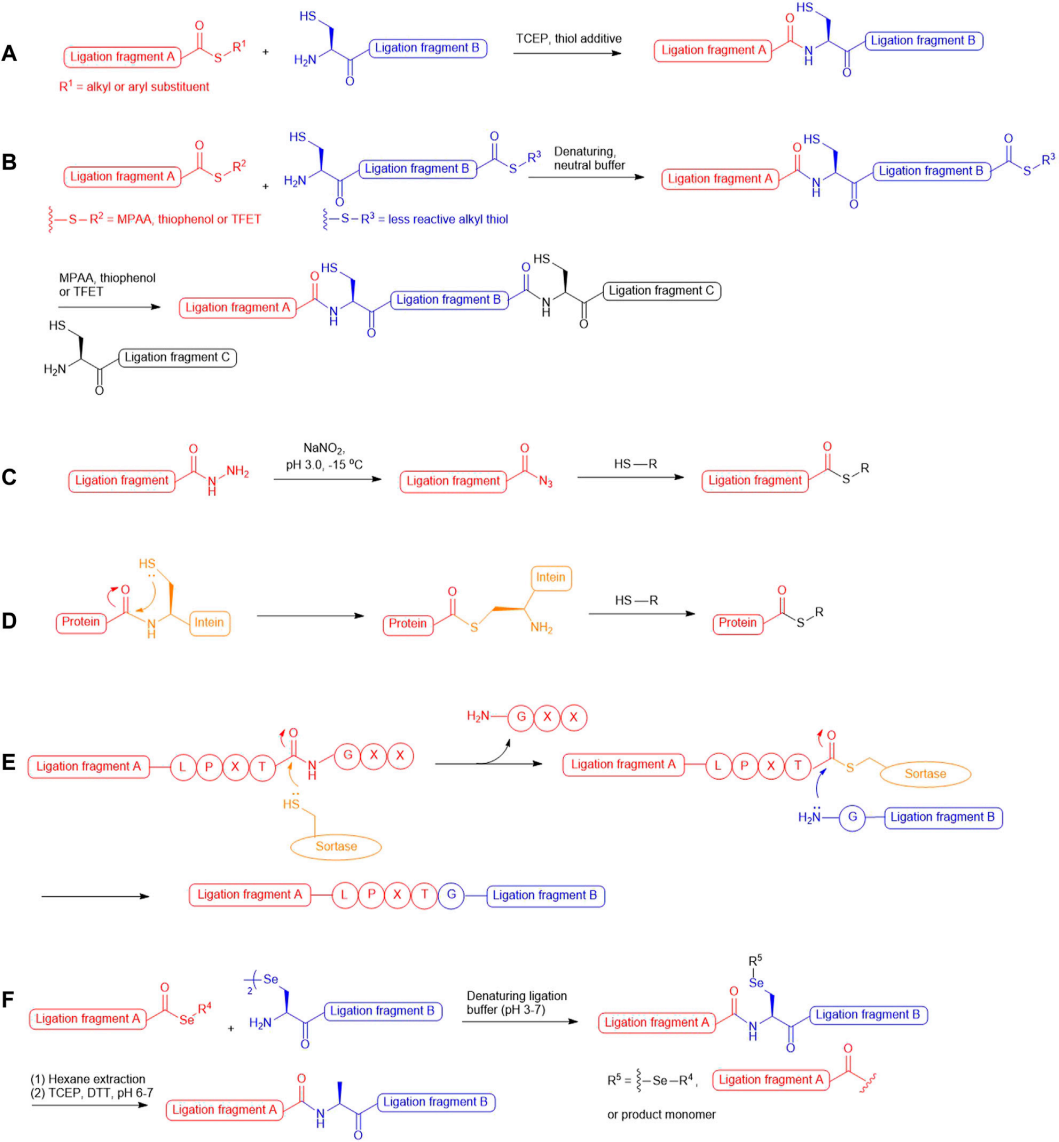
**(A)** Native chemical ligation; **(B)** Kinetically-controlled ligation;**(C)** Transforming peptide hydrazide to peptide thioester via oxidation and thiolysis; **(D)** Protein thioesterification via thiolysis of intein tag; **(E)** Sortase-mediated peptide ligation; **(F)** One-pot selenocystine-selenoester ligation-deselenization chemistry.

In the past two decades, native chemical ligation (NCL) technology has been broadly utilized for the convergent building of peptide and protein targets. As described in the seminal report by Kent and coworkers (Dawson et al., 1994), this technique involves chemical ligation of two unprotected peptide fragments bearing an N-terminal cysteine (Cys) and a C-terminal thioester respectively, and operates in neutral denaturing buffer to furnish the ligation product. The reaction starts with a reversible *trans*-thioesterification reaction through nucleophilic attack on the peptide thioester by the N-terminal Cys thiolate, leading to the formation of a bridged thioester intermediate that subsequently undergoes irreversible, intramolecular S-to-N acyl shift to afford a native peptide bond (Scheme 1A). Kent also demonstrated that sequential NCL reactions can be achieved by harnessing the differential reactivity of peptidyl thioesters (Bang et al., 2006): a bifunctional peptide fragment possessing an N-terminal Cys and a dormant alkyl thioester on the C terminus was ligated with a peptide phenyl thioester without the concern of head-to-tail peptide cyclization; aryl thiol additives [i.e., thiophenol and 4-mercaptophenylacetic acid (MPAA)] were added subsequently to activate the inert thioester which allowed the next NCL reaction to proceed *in situ* (Scheme 1B). This sequential ligation concept has been furthered by the use of 2,2,2-trifluoroethanethiol (TFET) to enhance the rate of peptide ligation through *in situ* generation of a productive TFET thioester (Thompson et al., 2014; Thompson et al., 2017). Peptide hydrazides have also been extensively used in iterative peptide ligation strategies as latent thioesters (Li et al., 2018; Fang et al., 2011). Importantly, the acyl hydrazide moiety is unresponsive to NCL conditions and acts as a protecting group for the C-terminal carboxylate, which can be converted to acyl azide by NaNO_2_ followed by thiolysis to unleash the capacity for NCL (Fang et al., 2011; Qi et al., 2019) (Scheme 1C). Recently, Flood *et al.* demonstrated that peptide hydrazides can be activated by a stoichiometric amount of acetyl acetone to produce peptide acyl-pyrazoles that will readily undergo transesterification reactions with aryl thiols to afford activated peptide thioesters for peptide ligation (Flood et al., 2018).

These powerful methodologies have been implemented in total synthesis of numerous protein targets and have demonstrated excellent compatibility with those bearing delicate post-translational modifications (PTMs), such as phosphorylation (Shogren-Knaak et al., 2003; Pan et al., 2019; Kilic et al., 2018; Jbara et al., 2016), sulfation (Watson et al., 2018; Hsieh et al., 2014), ubiquitination (Li et al., 2018; Yang et al., 2009; Ajish Kumar et al., 2009; Kumar et al., 2010; Kumar et al., 2011; Siman et al., 2013) and glycosylation (Wang et al., 2013; Wilkinson et al., 2012; Murakami et al., 2016). Whilst recombinant expression technologies remain important means to access large and structurally-complex proteins, they cannot afford the same level of target precision and chemical diversity that chemical protein synthesis provides. In particular, the enzymatic nature of PTM machineries in cells leads to target protein production as a heterogeneous mixture of different PTM forms that are unresolvable using chromatographic techniques. Examples that highlight the ability to access defined PTM forms of natural protein targets via NCL chemistry include the total synthesis of homogeneously glycosylated erythropoietin (Wang et al., 2013), different modified variants of histone proteins (Jbara et al., 2016; Siman et al., 2013; Seenaiah et al., 2015; Jbara et al., 2017) and sulfated anticoagulant proteins (Thompson et al., 2017; Watson et al., 2018; Hsieh et al., 2014; Watson et al., 2019). Despite our ability to perform chemical synthesis of post-translationally modified proteins using suitably protected Cys and acyl donors, the number of iterative ligation steps that can be performed on a large scale to provide the final target in sufficient quantity is often limited. This limitation has spurred tremendous interest in converging NCL with intein-mediated protein splicing technology referred to as Expressed Protein Ligation (EPL) (Muir et al., 1998). Harnessing the thiolytic nature of intein-based protein tags, this method allows for the generation of recombinant proteins possessing C-terminal thioesters (Scheme 1D), which permits further engineering with exogenous peptides bearing an N-terminal Cys residue. Readers are advised to refer to a plethora of excellent reviews (Conibear et al., 2018; Thompson and Muir, 2020; Wang and Cole, 2020; Muir, 2003; Flavell and Muir, 2009; Hofmann and Muir, 2002) that summarize the tremendous progress in the area of EPL. The NCL method has also been combined with chemoenzymatic ligation (Thompson and Muir, 2020; Nuijens et al., 2019; Schmidt et al., 2017; Weeks and Wells, 2020; Arnold, 2018) to access protein targets with residue-specific modifications, as exemplified by the recent semisynthesis of β2-adrenergic receptors reported by Liu and coworkers, which combines sortase-catalyzed ligation (Scheme 1E) with NCL to access discrete phosphorylation and ubiquitination forms (Li et al., 2021a).

The presence of an N-terminal Cys residue in one of two peptide fragments was a prerequisite for NCL chemistries, which significantly limited the possible disconnection routes in retrosynthetic analysis, particularly given the low occurrence of Cys among all the proteogenic amino acids (1.8%) (Moura et al., 2013). This synthetic barrier has attracted global research endeavours to expand the scope of NCL by developing new cysteine surrogates empowering the access to Cys-free protein targets and those having Cys residues at positions inadequate for peptide ligation. Initial attempts included the use of N-terminal thiol auxiliaries to mediate NCL reactions (Yan and Dawson, 2001; Offer, 2010), and recently, Li *et al.* also exploited the potential of glycyl auxiliary in isopeptide ligation with a view to precisely introduce ubiquitin modifications to the protein of interest (i.e., H2A) (Li et al., 2018). However, such peptide ligation strategies typically require long reaction times and additional steps to remove the auxiliaries, whereby hydrolyzed and epimerized by-products can develop over time and undermine the overall ligation yield. In this exploratory journey, one of the most effective approaches is to incorporate a thiol moiety on the side chain of the N-terminal amino acid that enables peptide ligation in a way analogous to that at Cys residues. The scope of such NCL methodology was expanded by Yan and Dawson through the development of a post-ligation desulfurization chemistry mediated by either Raney Ni or Pd, whereby traceless removal of the thiol appendage could be achieved (Yan and Dawson, 2001). However, the large excess of Ni or Pd used in the desulfurization reaction can result in undesirable tryptophan hydrogenation or methionine demethylthiolization. Later, Danishefsky and coworkers furthered this ligation−desulfurization concept through applying milder, metal-free radical desulfurization conditions to remove the thiol moiety, which consists of a water-soluble radical initiator 2,2ʹ-azobis [2-(2-imidazolin-2-yl)propane]dihydrochloride (VA-044), TCEP and a sulfhydryl donor in neutral ligation buffer (Wan and Danishefsky, 2007). Importantly, this radical desulfurization chemistry is orthogonal to PTMs on the side chains of proteogenic amino acids and peptidyl thioesters, which facilitates rational design of sequential, multi-component ligation strategies to access large proteins.

In parallel to the development of ligation-desulfurization methods, there have been considerable efforts to address other practical limitations, including the sluggish reaction rates at sterically encumbered ligation junctions and the lack of discrimination between unnatural thiolated amino acids and unprotected cysteine residues under desulfurization conditions. In 2001, three research groups demonstrated the utility of selenocysteine (Sec) in NCL-like reactions via nucleophilic attack on peptide thioesters followed by Se-to-N acyl shift to afford large selenopeptides and selenoproteins (Hondal et al., 2001; Quaderer et al., 2001; Gieselman et al., 2001). Another landmark report in Sec-mediated NCL methodology from Dawson and coworkers described chemoselective deselenization chemistry that proceeded smoothly to completion in the presence of free Cys residues when using excess amounts of TCEP and dithiothreitol (Metanis et al., 2010). The observed chemoselectivity has been attributed to the weaker C–Se bond favouring homolytic cleavage via the reaction between phosphine and a selenium-centered radical, which results in the generation of a phosphine selenide intermediate and alanyl radical that undergoes hydrogen atom abstraction from a donor to regenerate an alanine residue. The resulting ligation−deselenization method opened a new pathway to access proteins bearing structurally and functionally important Cys residues. However, the selenol moiety in Sec possesses a strong reducing power and is oxidized rapidly in air to form the diselenide species (selenocystine), which necessitated the use of excess reducing agents (i.e., TCEP, MPAA and thiophenol) to maintain a productive level of selenol for NCL reactions. Recently, Payne and coworkers reported that utilizing aryl selenoesters as the ligation counterparts of selenocystine can provide substantial enhancement in ligation rates, leading to clean ligation within minutes even at the sterically demanding junctions (Mitchell et al., 2015). Remarkably, this method involves the simple mixing of peptide selenoesters with the peptide diselenide dimer in ligation buffer without the need of reducing agents (Scheme 1F). The additive-free reaction dubbed diselenide–selenoester ligation (DSL) represents an unprecedented reactivity of Sec that gateways to rapid generation of polypeptide and protein therapeutic leads bearing unique and defined modifications (Watson et al., 2018; Watson et al., 2019; Agten et al., 2021). These pioneer works have also fuelled the development of synthetic routes to assess suitably protected β/γ/δ-selenyl derived amino acids that can be employed in traceless selenium-mediated peptide ligation for total synthesis of protein targets bearing distinct modifications. This review will highlight the contributions of thiolated and selenylated amino acid synthesis in the advancement of ligation methodologies for detailed studies on protein structure and function influenced by PTMs.

## Synthesis of Thiolated/Selenylated Amino Acids

### β-Thiol/selenol Derived Leucine

**SCHEME 2:**
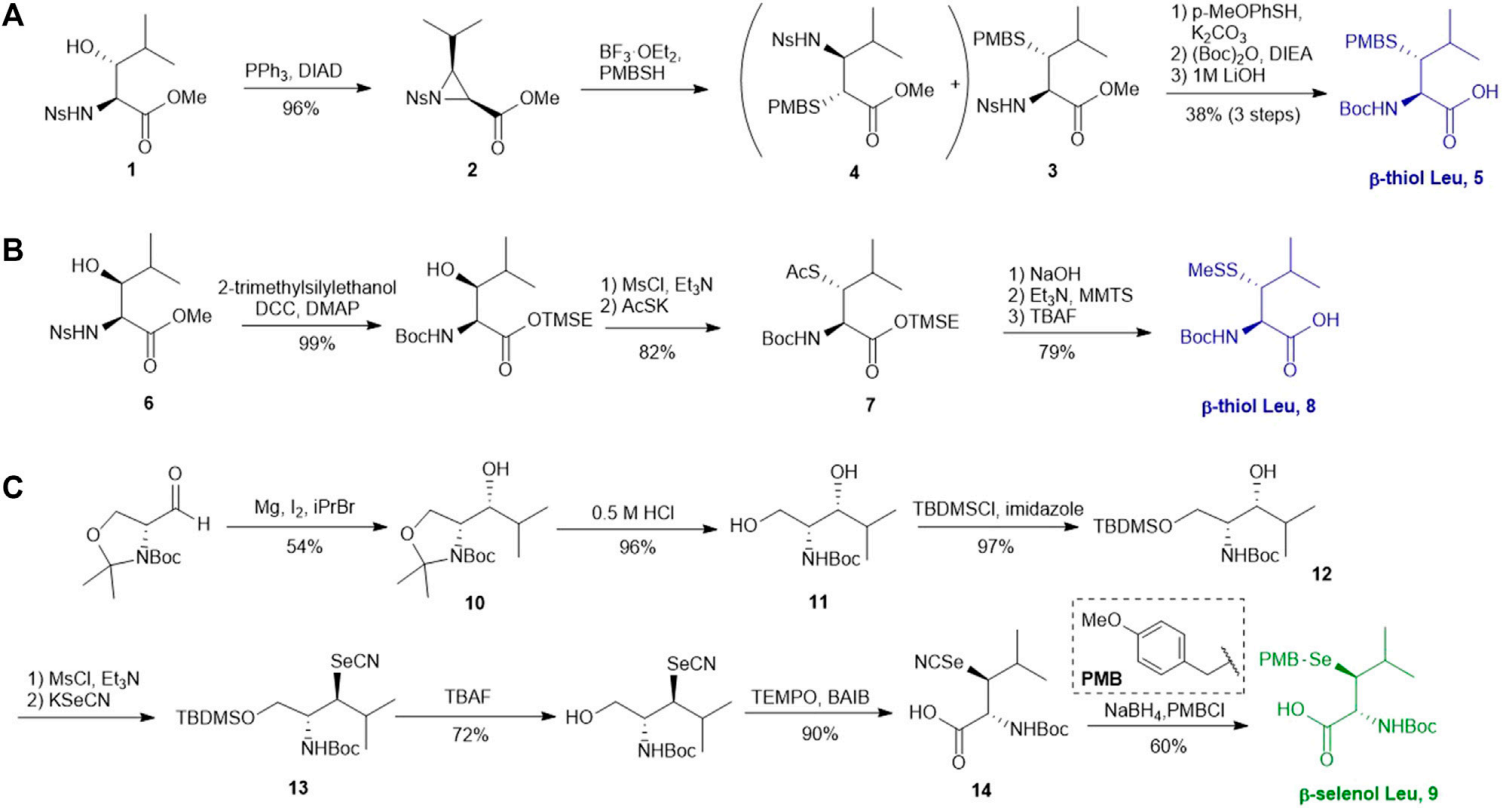
**(A)** Preparation of β-thiol Leu via Brik’s route; **(B)** Preparation of β-thiol Leu via Danishefsky’s route; **(C)** Synthesis of β-selenol Leu via Payne’s route.

Several β/γ-hydroxyl amino acids are commercially available as ideal starting materials for thiolated amino acid synthesis. For example, β-hydroxyleucine has been implemented in the synthesis of β-thiol Leucine (Leu) by Brik’s and Danishefsky’s groups (Harpaz et al., 2010; Tan et al., 2010) (Scheme 2A). Brik and coworkers introduced a nosyl group to the α-amine moiety of a hydroxyl Leu to form **1**, facilitating the aziridine formation via an intramolecular Mitsunobu reaction to afford **2** (Harpaz et al., 2010). Ring-opening by PMB-SH in the presence of BF_3_ as Lewis acid afforded the β-thiolated Leu intermediate (**3**) and its regioisomer (**4**) in a 6:4 ratio, which are separable by column chromatography. This reaction was stereospecific, directed by the α-chiral center to produce *anti*-product exclusively. After three steps of protecting group manipulation, the desired *p*-methoxybenzyl (PMB)-protected β-thiol Leu (**5**) was furnished in a 38% yield. The PMB protecting group is stable under fluorenylmethoxycarbonyl-based solid phase peptide synthesis (Fmoc-based SPPS) conditions, acidolytic deprotection and cleavage conditions; it can be readily removed using Hg (OAC)_2_ and DTT in trifluoroacetic acid (TFA) before ligation.

Danishefsky and coworkers demonstrated a different route to β-thiol Leu, where the hydroxyl group on the side chain of the protected β-hydroxy-Leu (**6**) was activated through mesylation and displaced by thioacetate (Scheme 2B) (Tan et al., 2010). Interestingly, direct thiol substitution using thioacetic acid or PMB-SH as nucleophile led to substantial β-elimination product, while the conjugate base of thioacetic acid can mitigate the formation of the elimination by-product. This approach gave the *anti*-product **7** exclusively (in 82% yield over two steps) due to the intrinsic β-chiral center of the amino acid. Next, the thioacetate moiety was transformed to methyl disulfide after saponification and oxidation with methane methylthiosulfonate (MMTS), and the carboxylic acid was unmasked using tetra-n-butylammonium fluoride (TBAF) to provide the desired β-thiol Leu building block (**8**) for SPPS. This approach served as a synthetic template to access β/γ-thiol amino acids, allowing one to access a single stereoisomer to study the impact of the β/γ-chiral centers on peptide ligation efficiency. For example, peptides possessing an *anti*-β-thiolated Leu at the N-terminus can efficiently react with peptide thioesters terminated at Phe residues, however sluggish ligation reactions were observed when using the *syn*-isomer (Tan et al., 2010). The underlying mechanism has been proposed: the *syn*-conformation formed in the process of transesterification causes steric congestion between the isopropyl side chain and peptide backbone that disfavors the S-to-N acyl shift. The β-thiol Leu **7** has also been masked by a thiazolidine protecting group via acidolysis followed by paraformaldehyde incubation. The resulting β-thiol Leu has been used in the convergent synthesis of the ATAD2 bromodomain region (Creech et al., 2014).

Wang *et al.* demonstrated the use of Garner’s aldehyde to access selenylated Leu **9** (Scheme 2C) (Wang et al., 2017). The synthesis began with the Grignard addition of iPrMgBr to Garner’s aldehyde resulting in the formation of oxazolidine **10** as a single diastereomer. Subsequent nucleophilic displacement of the *syn*-mesylate generated from **10** by KSeCN was unsuccessful due to the unfavourable conformation imposed by the oxazolidine ring. The isopropylidene moiety was thereby removed to provide the diol **11** followed by selective protection of the primary alcohol with TBDMS ether (**12**). The resulting secondary alcohol underwent mesylation and substitution smoothly to afford the *anti*-selenylated product **13**. After protecting group manipulation and TEMPO-BAIB oxidation to yield the amino acid **14**, the selenocyanate moiety was reduced and subjected to PMB protection to furnish the desired selenyl Leu **9** in a 60% isolated yield.

### γ-Thiol/selenol Derived Proline

**SCHEME 3:**
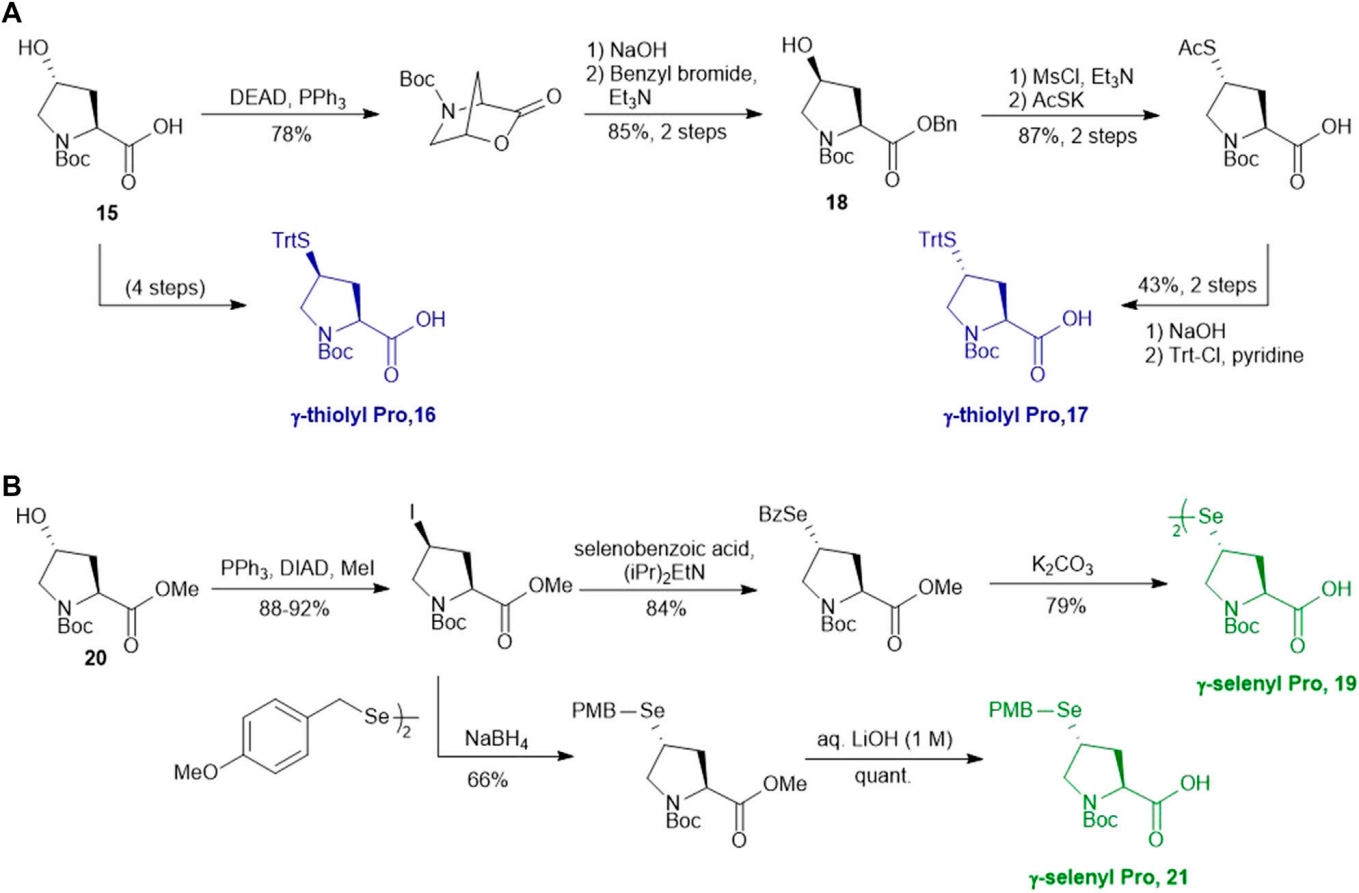
**(A)** Synthesis of γ-thiol derived Pro; **(B)** Synthesis of γ-selenylated Pro.

Compared to β-hydroxyl Leu, γ-hydroxyl proline (Pro) building blocks are more readily available, i.e., USD $3 per Gram of the Boc-protected *trans-*γ-hydroxyl Pro (**15**). Through mesylation and displacement by thioacetate, a *syn*-thiol Pro (**16**) can be readily prepared at a large scale (Ding et al., 2011). To afford *anti*-thiol Pro **17**, *syn*-hydroxyl Pro **18** was firstly produced via an intracellular Mitsunobu reaction followed by saponification (Scheme 3A). The carboxylic acid was then protected before thioacetate displacement to afford *anti*-thiolated Pro **16**. Interestingly, only the *anti*-isomer manifested satisfying reactivity in NCL reactions. The rat neuromedin U had been successfully prepared through *anti*-thiol Pro-mediated ligation followed by desulfurization.

Though the thiolated Pro **17** had been applied to the chemical protein synthesis of complex glycosylated proteins, its ligation efficiency was severely compromised by the presence of sterically hindered thioesters. In order to enhance the rate of inter- and intramolecular acyl transfer in Pro ligation, *anti*-selenylated Pro **19** was also prepared by Danishefsky and coworkers (Scheme 3B) (Townsend et al., 2012). The synthesis began with Mitsunobu inversion of the hydroxyproline **20** to provide a *syn*-iodoproline intermediate followed by nucleophilic displacement with selenobenzoic acid and hydrolysis to afford the desired diselenide **19** in a 85% yield over two steps. The authors first examined the ligation efficiency of **19** under standard NCL conditions with excess MPAA as a thiol additive. MPAA served to activate the alkyl thioester through transthioesterification; it also reduced the selenium dimer, liberating a small amount of productive selenol to mediate the peptide ligation. Under these conditions, model ligation reactions were completed within 3 h in good to excellent yields. The relative ease of deselenization compared to desulfurization in model peptides were also demonstrated using Dawson’s conditions (DTT, TCEP, ligation buffer, pH 5.0–6.0) (Townsend et al., 2012).

Recently, Sayers *et al.* demonstrated the capacity of *anti*-γ-selenyl Pro **21** in rapid DSL reactions (Sayers et al., 2018a) (See Section 4). Initially, they followed Danishefsky’s route to prepare γ-selenyl Pro building block for SPPS. However, to overcome the issue of having only one Pro unit within the diselenide dimer being coupled to resin-bound peptides, monomeric γ-selenyl Pro **21** was prepared instead, wherein the γ-selenol moiety was masked with a PMB group to prevent oxidative dimerization. This building block can be efficiently incorporated into the N-termini of resin-bound model peptides using only a slight excess of **21** (1.2 equiv.) together with hydroxyazabenzotriazole (HOAt) (1.2 equiv.), N,N′-diisopropylcarbodiimide (DIC) (1.2 equiv.). Acidolysis of side-chain protecting groups with concomitant peptide cleavage from the resin was followed by deprotecting the PMB group in 20% DMSO, 5% iPr_3_SiH in TFA. Purification by HPLC provided the desired peptide diselenide dimer in 50% overall yield.

### β-Thiol/selenol Derived Phenylalanine

**SCHEME 4:**
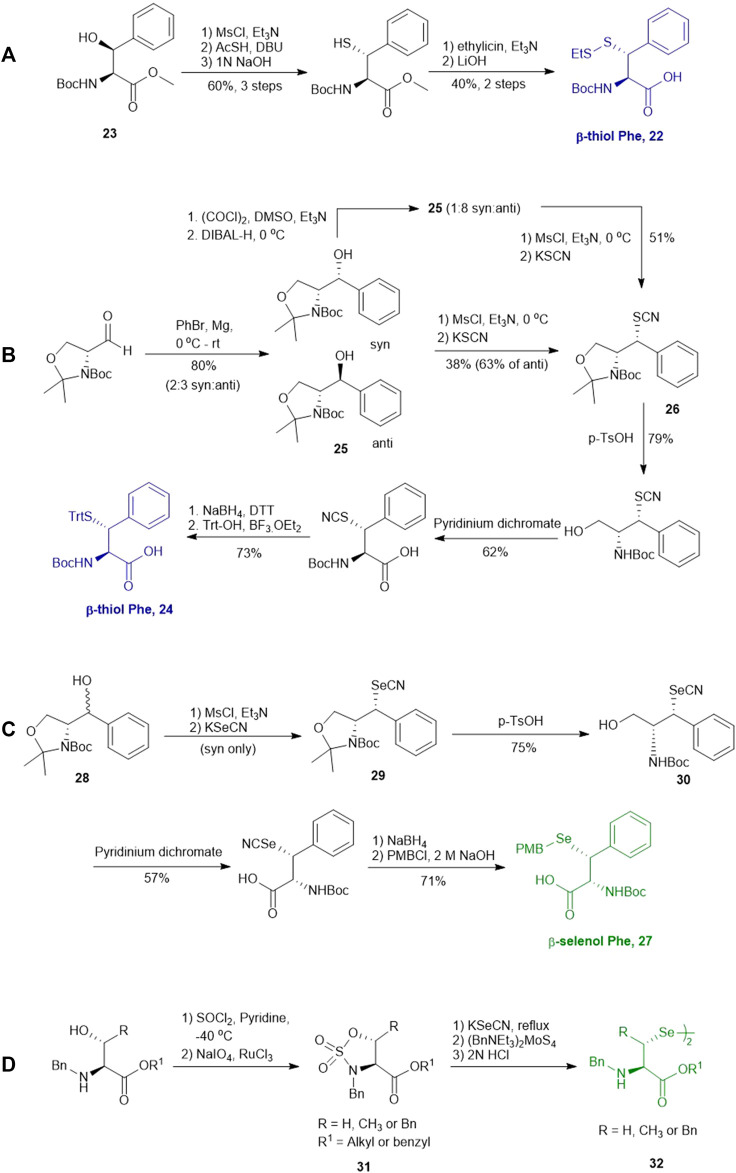
**(A)** Synthesis of β-thiolated Phe via Easton's route. **(B)** Synthesis of β-thiolated Phe via Payne's route. **(C)** Synthesis of β-selenylated Phe reported by Malins et al.; **(D)** Asymmetric synthesis of β-selenylated Phe via a cyclic sulfamidate intermediate reported by Baig et al.

In cases where β/γ-hydroxyl amino acids are not commercially available, the thiolated amino acid can be prepared from the corresponding proteogenic amino acids. Banerjee and coworkers began the synthesis of β-thiol phenylalanine (Phe) **22** by preparing the (2*S*,3*S*)-hydroxyl Phe **23** according to Easton’s protocol (Scheme 4A) (Easton et al., 1994). The following synthesis was performed in analogy to the sulfenylation route mentioned above (via a mesylation−nucleophilic displacement manifold), affording the desired *anti*-β-thiol Phe **22**. The utility of **22** in chemical protein synthesis has also been demonstrated by the authors (Crich and Banerjee, 2007).

Payne and coworkers harnessed Garner’s aldehyde to build a hydroxylated Phe precursor in order to access β-thiol Phe **24** (Scheme 4B) (Malins et al., 2015a). Firstly, the phenyl alcohol (**25**) was formed in the reaction between a Grignard reagent of bromobenzene and Garner’s aldehyde as a mixture of two diastereomers in an excellent yield (80%). The moderate stereoselectivity is due to two competing mechanisms for asymmetric induction: the *anti*-configured isomer is postulated to proceed through the Felkin-Ahn transition state, whereas in the presence of Mg cations, the reaction can proceed via a Cram’s chelation transition state resulting in the *syn* product (Passiniemi and Koskinen, 2013). Next, the alcohol was mesylated followed by nucleophilic displacement by KSCN to afford the desired product in a yield of 38%. Only the *anti*-isomer of **25** could proceed to completion, which provided an opportunity to resolve the diastereomeric mixture by chromatographic purification. Notably, the unreactive *syn*-isomer of **25** was inverted via Swern oxidation followed by asymmetric reduction with DIBAL to provide more of the *anti*-isomer (Malins et al., 2015a). The oxazolidine moiety in **26** was removed using *p-*toluenesulfonic acid in dioxane to obtain the free alcohol in a good yield (79%). This alcohol was then oxidized with pyridinium dichromate to afford the carboxylic acid in a reasonable yield (62%). Finally, the thiocyanate was reduced and masked with a trityl (Trt) protecting group to afford the desired β-thiolated amino acid in 73% yield (Malins et al., 2015a).

Malins *et al.* reported the use of a similar strategy to access β-selenylated Phe (**27**), which started with the Grignard addition of phenylmagnesium bromide to Garner’s aldehyde to provide **28** as an inseparable mixture of *syn* and *anti*-diastereoisomers (Scheme 4C) (Malins and Payne, 2012). This mixture was used in the next step, which involved mesylation of the secondary alcohol followed by nucleophilic displacement with potassium selenocyanate to afford the oxazolidine **29**. Notably, the isomers could be resolved by chromatography at this stage; only the *anti*-mesylate was active in the substitution reaction leading to the desired *syn*-selenocyanate (**29**). Acid deprotection of the oxazolidine **29** followed by oxidation with pyridinium dichromate yielded the carboxylic acid **30** in moderate yield without any sign of selenium oxidation. Finally, the selenocyanate moiety was reduced with sodium borohydride followed by protection with 4-methoxybenzyl chloride to afford the desired β-selenylated Phe (**27**) in a good yield (72% over two steps).

Baig *et al.* reported the asymmetric synthesis of several cyclic sulfamidate from the respective β-hydroxyl amino acids (Scheme 4D) (Rashid Baig et al., 2010). A benzyl sulfamidate (**31**) was transformed to β-selenylated Phe **32** through nucleophilic displacement with KSeCN followed by reductive dimerization mediated by tetrathiomolybdate.

### Thiol/Selenol Derived Lysine

**SCHEME 5:**
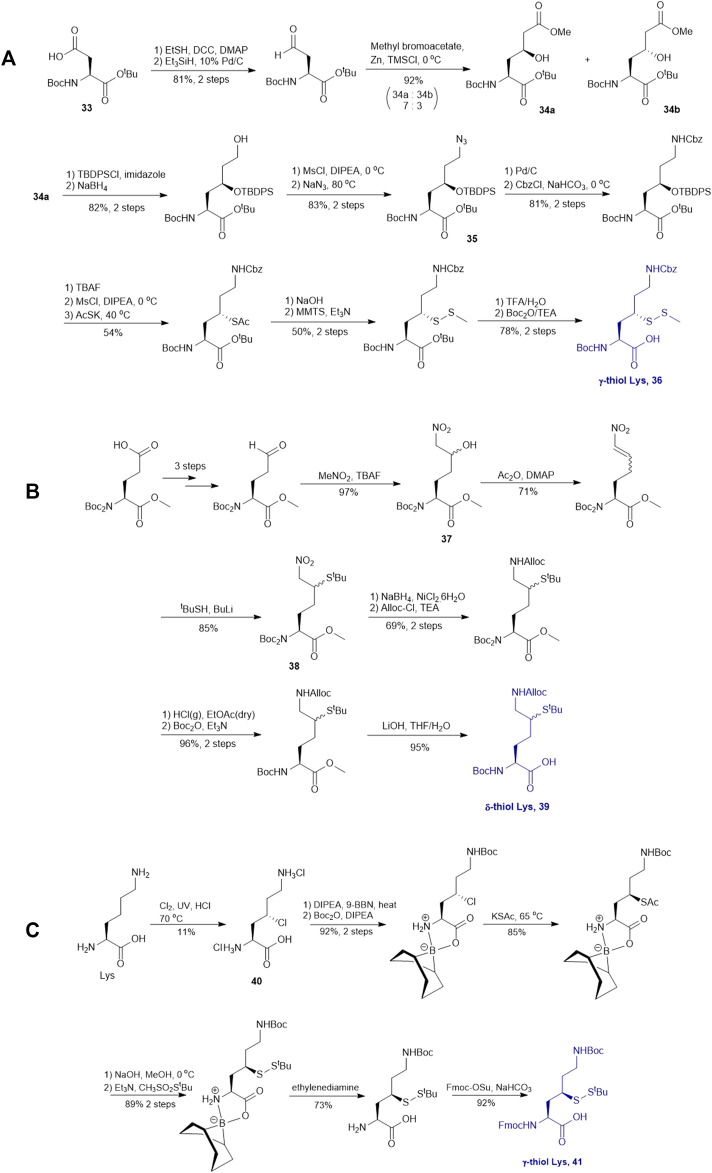
**(A)** Synthesis of γ-thiolated Lys reported by Liu and co-workers; **(B)** Synthesis of δ-thiolated Lys via Brik's route; **(C)** Synthesis of γ-thiolated Lys reported by Merkx et al.

γ-thiol lysine (Lys) was first synthesized by Liu and coworkers, using Asp as the precursor (Yang et al., 2009; Marin et al., 2004). Subsequently, they proposed a general, diastereoselective approach to prepare γ-thiol Lys derivatives (Scheme 5A) (Pasunooti et al., 2009). The side-chain carboxylate group of Asp **33** was first reduced to aldehyde, which allowed for asymmetric Reformatsky reaction to extend the side chain, leaving a hydroxyl group on the γ-carbon, followed by protection with *tert*-butyldiphenylsilyl group. The ester moiety in **34a** was reduced to alcohol that was subsequently activated and displaced by sodium azide to form **35**. The resulting azide group was reduced to form the ε-amine of Lys and protected with benzyl chloroformate. Finally, the γ-hydroxyl group was deprotected for sulfenylation through the mesylation−nucleophilic displacement manifold. This synthesis required 21 steps overall (including Asp protection), but a γ-thiol Lys building block (**36**) with defined stereochemistry could be achieved. The utility of **36** was demonstrated in the synthesis employing dual native chemical ligation to assemble ubiquitinated peptides, which will be further discussed in Section 4.

Brik and coworkers introduced a thiol functionality to the δ-carbon of Lys through conjugate addition (Scheme 5B) (Ajish Kumar et al., 2009). The side-chain carboxylate of Boc-Glu-OMe was first reduced to aldehyde followed by a Henry reaction to afford a *syn* and *anti* mixture of nitro alcohol **37**. The alcohol was acetylated and eliminated to provide a mixture of *trans*- and *cis*-nitro olefin as the Michael acceptor, which underwent electrophilic addition to *tert*-butylthiol to form a δ-thiolated Lys precursor (**38**) as a mixture of *syn- and anti-*isomers. The nitro moiety was reduced to the primary amine using a mixture of sodium borohydride and nickelous chloride, followed by protecting group modifications to afford the desired δ-thiol Lys (**39**).

Alternatively, thiolated Lys has been synthesized through the conversion of Lys to γ-chlorinated Lys **40** in concentrated hydrochloric acid under UV irradiation with continuous chlorine gas bubbling (Merkx et al., 2013; Van Der Heden Van Noort et al., 2017) (Scheme 5C). Despite low yield (11%), the reaction is highly stereospecific and regioselective. The α-amine and carboxylic acid of γ-chlorinated Lys (**40**) was subsequently masked by 9-BBN while the side-chain amine was protected with Boc anhydride. This was followed by the nucleophilic displacement of the chloride substituent by thioacetate (Merkx et al., 2013). Final protecting group manipulation afforded the desired γ-thiol Lys building block **41** for Fmoc-based SPPS.

**SCHEME 6:**
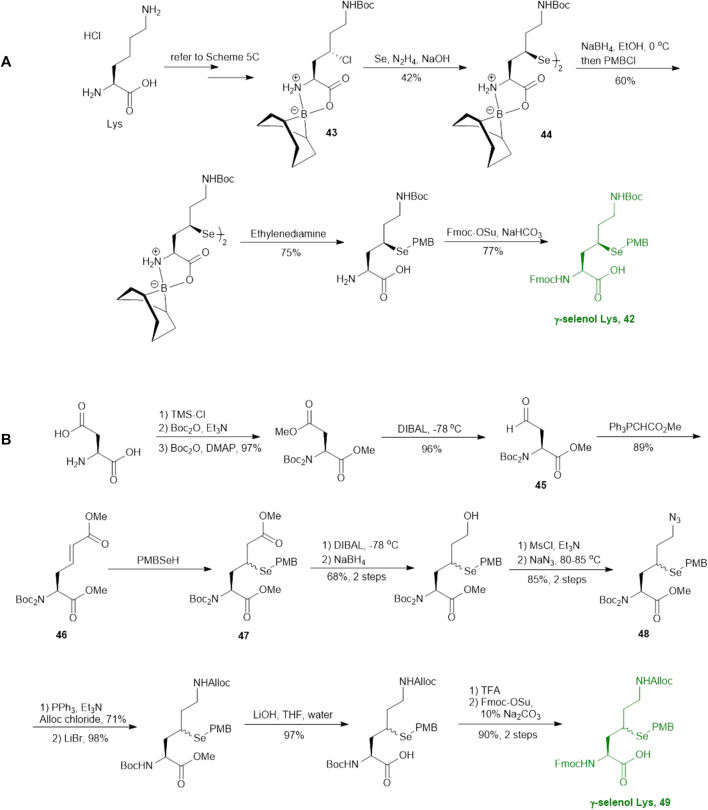
Synthesis of two differentially functionalized γ-selenol Lys reported by Metanis and co-workers.

As will be discussed in the later section, γ/δ-thiolated Lys has been frequently used in isopeptide ligation to facilitate site-specifical incorporation of (poly) ubiquitin modifications into small proteins. However, this strategy poses a challenge in EPL, where a specialized expression strategy is required to incorporate protecting groups for cysteine residues native to the protein of interest. Therefore, the Metanis group sought to develop a γ-selenylated Lys that enables selenium-mediated isopeptide ligation followed by using site-specific deselenization chemistry to furnish the native Lys in the presence of unprotected Cys residues (Scheme 6A) (Dardashti et al., 2020). They reported the synthesis and application of γ-selenyl Lys **42** as a tool molecule in an isopeptide ligation-deselenization manifold as such, although the preparation of δ-selenyl Lys was not successful due to spontaneous selenium elimination. The synthetic route toward γ-selenyl Lys **42** started with preparing the suitably protected γ-chlorinated Lys **43** following the protocol reported by Merkx *et al.* (Merkx et al., 2013). Nucleophilic displacement by Na_2_Se_2_ was performed to generate **44** in moderate yield, in which the selenol moiety was subsequently masked by PMB group. Finally, unmasking the α-amine and α-carboxylate with ethylenediamine followed by Fmoc-protection gave the desired selenylated building block **42** for SPPS in 77% yield.

In parallel to this synthesis, the authors also set out to synthesize a γ-selenyl Lys analogue having an orthogonally protected ε-amine such that dual chemical ligation can be performed on the same residue, whereby the γ-selenol will first assist with constructing the main peptide chain *via* NCL followed by isopeptide ligation to append the ubiquitin protein to the ε-amine (Scheme 6B) (Dardashti et al., 2020). The authors were unable to incorporate an orthogonal protecting group on the ε-amine beginning with the same γ-chlorinated Lys building block; therefore, an alternative route was developed using aldehyde **45** as the starting material, which can be prepared from L-Asp. The aldehyde **45** was extended with a triphenylphosphorane reagent to afford the *trans*-ester **46** in 85% yield over two steps, which was subjected to conjugate addition reaction with PMB selenol generated *in situ* to provide compound **47** as a mixture of two stereoisomers. The side-chain ester was selectively reduced to a primary alcohol, which was subsequently activated and displaced by sodium azide to afford **48**. Staudinger reduction of azide **48** followed by protecting group manipulation provided the desired γ-Se-Lys **49** was afforded in 90% isolated yield over two steps.

### γ-Thiol Derived Valine, Threonine and Isoleucine

**SCHEME 7:**
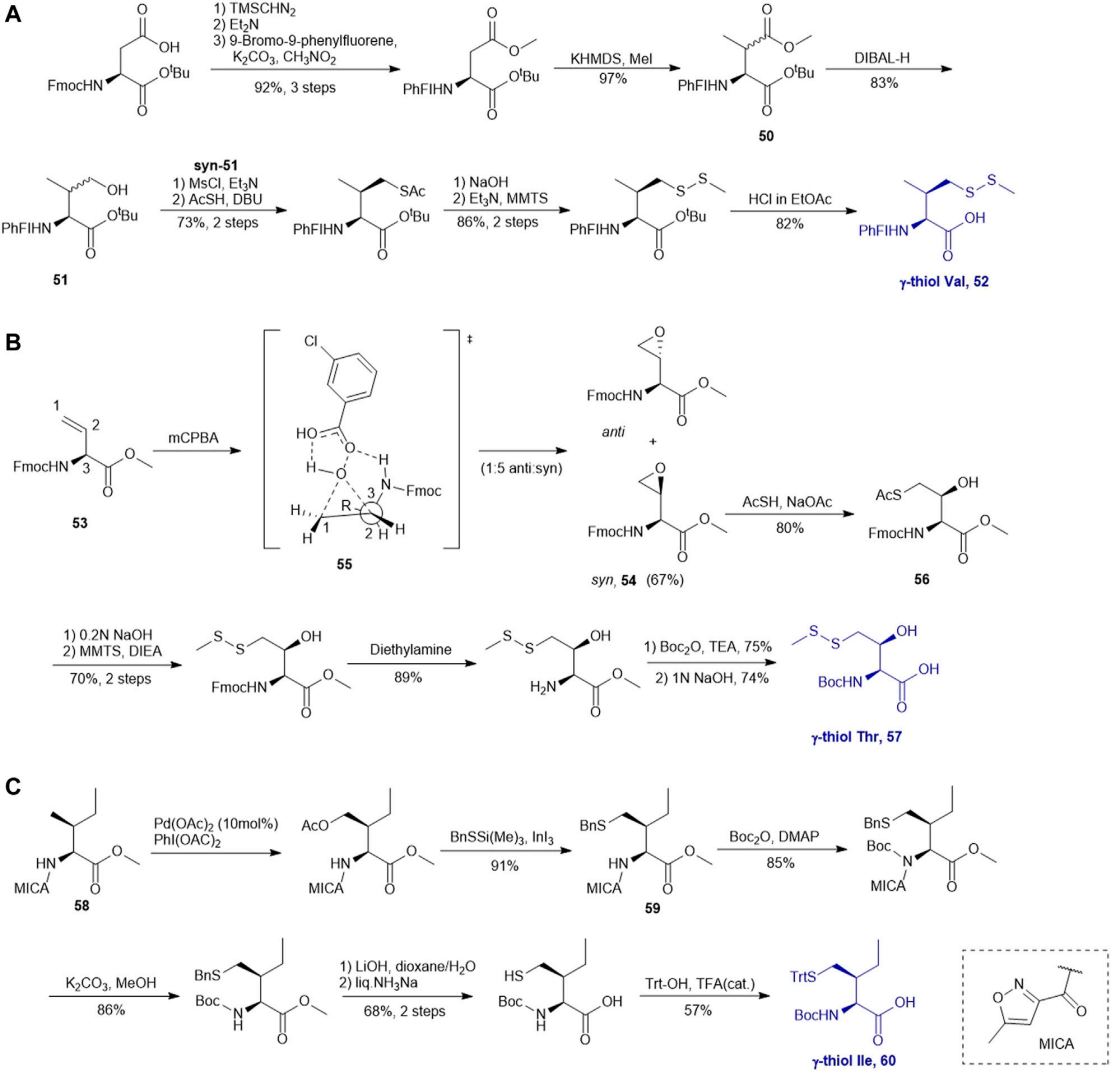
**(A)** Synthesis of γ-thiol derived Val; **(B)** Synthesis of γ-thiol derived Thr; **(C)** Synthesis of γ-thiol derived Ile.

A suitably protected Asp has been used in the synthesis of γ-thiol valine (Val) (Scheme 7A) (Chen et al., 2008). It began with introducing a [9-(9-phenylfluorenyl)] protecting group (PhFl) to the α-amine, which stabilized the α-chiral center and allowed for selective β-methylation leading to the amino acid **50** as a 1:1 mixture of *anti-* and *syn*-isomers. Selective reduction of the methyl ester produced γ-hydroxyl Val (**51**); and the *anti*- and *syn*-isomers were separated by chromatography. Finally, following the mesylation-nucleophilic displacement method, γ-thiol Val **52** was afforded in a good isolated yield (51% over five steps).

Vinylglycine (**53**) has been harnessed for the synthesis of γ-thiol threonine (Thr) through the formation of the *syn* epoxide intermediate **54** (Chen et al., 2010). Stereoinduction of the epoxidation reaction is attributed to hydrogen-bond stabilization of the peroxide transition state (**55**) by the α-amine (Scheme 7B). The epoxide ring was subsequently opened by thioacetate to provide the Thr intermediate (**56**) in the native configuration bearing a γ-thioacetate moiety. The final γ-thiol derived Thr **57** was afforded through several steps of protecting group manipulation. The reactivity of γ-thiol Thr has been demonstrated in model ligation studies using peptides bearing different C-terminal thioesters.

It is challenging to sulfenylate amino acids bearing hydrophobic or chemically unreactive side chains due to the lack of a directing group to facilitate the reaction. In 2015, Liu and coworkers had demonstrated a unique γ-C (sp_3_)-H functionalization strategy using 5-methylisoxazole-3-carboxamide (MICA) as a directing group to activate γ-carbon (Pasunooti et al., 2015). This strategy had been successfully applied to synthesize γ-thiol isoleucine (Ile) for the first time (Pasunooti et al., 2016) (Scheme 7C). γ-thiol Val and γ-thiol Thr were also synthesized from their natural amino acid counterparts following a similar synthetic route. MICA was first introduced to the α-amine of a Ile amino acid to form **58**, which directed the subsequent γ-acetoxylation. It was proposed that the MICA group first complexes with Pd^II^, which is subsequently oxidized to Pd^IV^ by PhI (OAC)_2_. The resulting complex proceeded through an octahedral transition state that exhibited an out-of-plane conformation favoring acetoxylation at the methyl branch of Ile (Buettner et al., 2019; Shabani et al., 2021). The resulting acetoxyl group was directly displaced by benzylthiol to form **59** in the presence of an indium triiodide catalyst (Nishimoto et al., 2012). The N-terminus of γ-thiol amino acid required Boc-protection before removing MICA using K_2_CO_3_ in MeOH. The final γ-thiol amino acid **60** was furnished after protecting group alternation. The utility of γ-thiol Ile (**60**) in peptide ligation has been demonstrated in the chemical synthesis of *Xenopus* H3.

### Thiol/Selenol Derived Aspartate, Glutamate and Asparagine

Multiple thiolated amino acids have been synthesized *via* electrophilic sulfenylation chemistry due to its concise, direct approach to installing a thiol functional group on nucleophilic carbon in the side chain of amino acids. This approach has been proven effective in the synthesis of thiolated aspartic acid (Asp) and glutamic acid (Glu) with protected side chain esters that can be chemoselectively enolized for nucleophilic addition on an electrophilic sulfur center.

**SCHEME 8:**
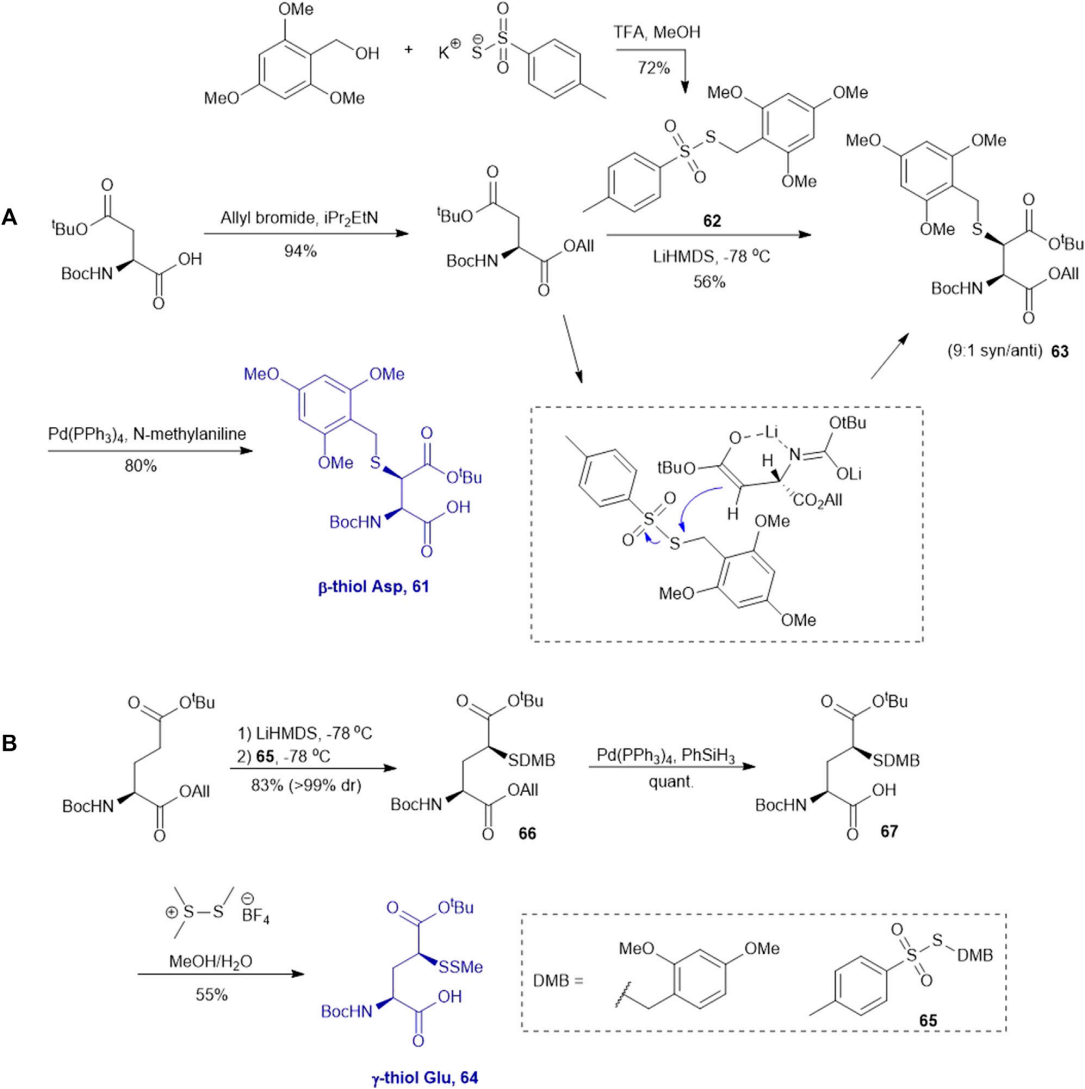
Synthesis of **(A)** β-thiolated Asp and **(B)** γ-thiolated Glu via electrophilic sulfenylation chemistry.

This approach was first conducted by Payne and coworkers in 2013 for the synthesis of β*-*thiol derived Asp **61**. (Thompson et al., 2013). They first prepared S-(2,4,6-trimethyoxybenzyl)toluenethiosulfonate (**62**), from 2,4,6-trimethylbenzyl alcohol and potassium toluene thiosulfonate (Scheme 8A). Using this sulfenylating reagent, the subsequent synthesis of Boc-Asp (O^*t*^Bu)-OH was conducted in three steps. Firstly, the free carboxylic acid was protected using allyl bromide (94%), then subjected to sulfenylation conditions, wherein the side-chain carboxylate ester was first enolized to form a lithium complex followed by nucleophilic attack at the electrophilic sulfur center of the sulfenylating reagent **62** at a low temperature leading to the formation of thiolated amino acid **63**. This reaction exploits the native α-chiral center to induce stereoselectivity. It proceeds through a 6-membered transition state where the Li coordinates with the enolate oxygen and protected nitrogen (Scheme 8A insert). This allows for preferential attack on the less hindered face, allowing for stereochemical control in favor of the *syn* configured diastereomer (9:1 *syn/anti*) (Shibata et al., 1996). The two diastereomers were separable by column chromatography to provide a stereochemically pure product in 56% yield. Finally, the allyl ester was deprotected using palladium tetrakis triphenylphosphine to obtain the desired thiolated amino acid **61** in an 80% yield (Thompson et al., 2013). The reactivity of β-thiol Asp was first evaluated in model ligation studies with various thioesters. These ligation reactions were conducted under standard NCL conditions and proceeded with high yields. They were also converted to the native peptides smoothly via Danishefsky’s desulfurization approach. A one-pot ligation−desulfurization method was also developed with this amino acid. This approach also addressed the limitation of lacking chemoselective desulfurization chemistries in the presence of native, unprotected Cys residues. First the ligation was performed in identical denaturing conditions and the aryl thiol was subsequently removed by ether extraction upon ligation completion. TCEP and DTT were then added to initiate a desulfurization reaction occurring on the β-carbon at pH 3, 65^o^C for 20 h with reasonable yields ranging from 48–63%.

Electrophilic sulfenylation chemistry has also been applied to the synthesis of γ-thiolated Glu **64** through enolization of the side-chain carboxylate ester and sulfenylation *in situ* with **65** to afford **66** in a 83% isolated yield as a single diastereomer (Cergol et al., 2014). Product **66** was subjected to allyl deprotection conditions to afford the free acid **67** in quantitative yield (Cergol et al., 2014). This DMB protected thiolated amino acid **67** was incorporated into the N-terminal position of a model amino acid, however after acidolytic cleavage and deprotection, no desired product was isolated. The authors rationalized that acidolytic deprotection of the DMB group liberated the γ-thiol that could undergo nucleophilic attack at the amide bone leading to the deletion of this thiolated residue (Cergol et al., 2014). Therefore, the DMB protecting group was switched to an acid-stable but reductively labile methyl disulfide protecting group, which allowed for stable incorporation into peptide N-termini for NCL reactions (Scheme 8B). The utility of **64** in NCL reactions has been demonstrated in model ligation studies using a variety of peptide thioesters and in chemical synthesis of a known peptidic drug using a one-pot ligation−desulfurization method.

**SCHEME 9:**
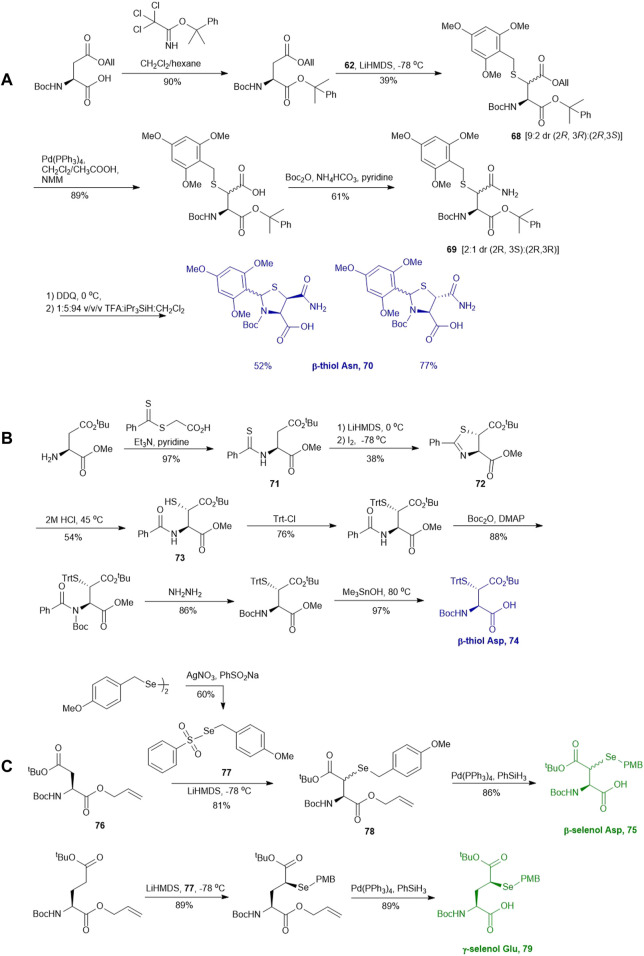
**(A)** Synthesis of β-thiol derived Asn via electrophilic sulfenylation chemistry; **(B)** Tan’s route to prepare β-thiol derived Asp; **(C)** Synthesis of β-selenol derived Asp and γ-selenol derived Glu via electrophilic selenylation chemistry.

Similar to the synthetic methods mentioned above, Payne and coworkers also prepared β-thiol asparagine (Asn) using similar electrophilic sulfenylation chemistry (Scheme 9A) (Sayers et al., 2015). They began with the readily available Boc-Asp (OAll)-OH which was protected with phenylisopropyl trichloroacetimidate to mask the α-carboxylate. The side-chain carboxylate was enolized by LiHMDS and reacted with the sulfenylating reagent **62** to form the thiolated derivate **68** as a mixture of two diastereomers (*syn*:*anti*, 9:2, Scheme 9A). The allyl ester was deprotected to liberate the free carboxylic acid, which was submitted to amination reaction containing Boc anhydride, ammonium bicarbonate, and pyridine to form the carboxamide side chain of **69**. The resulting *syn-* and *anti-*isomers were separable by column chromatography. From this, they attempted to directly couple this to a resin-bound peptide, however they found that lack of protection of the side chain led to quantitative succinimide formation. To suppress this side reaction, they used oxidative cyclization chemistry to form the final 2,4,6-trimethoxyphenyl-thiazolidine protected Asn **70** through generating a benzylic carbocation that underwent electrophilic addition onto the α-amine. This resulting building block was incorporated into peptides on resin smoothly, and was readily deprotected in 89:5:5:1 TFA:*i*Pr_3_SiH:H_2_O:EDT to liberate the free thiol for NCL chemistry (Sayers et al., 2015).

Another synthetic route to β*-*thiol derived Asp was recently developed by Tan and coworkers harnessing the chemistry of direct thiylation of a protected Asp (Scheme 9B) (Guan et al., 2013). They first prepared the thiobenzamide ester (**71**) using 2-​[(phenylthioxomethyl​)​thio]​-acetic acid and triethylamine in pyridine (97%). They then formed the *trans*-thioazoline in a two-step synthesis involving side-chain enolization using LiHDMS followed by electrophilic iodination of the β-carbon, which was subsequently attacked by the sulfur to form a thiazolidine (**72**) in 38% yield. Through this process, no *cis*-isomer was observed and stereochemistry was maintained throughout the remaining synthetic steps (Harpaz et al., 2010). Next, the thiazoline was hydrolyzed in 2M HCl to afford the N-benzoyl derivative (**73**) in 54% yield. Finally, protection of the free thiol with a trityl group together with other protecting group transformation reactions furnished the desired building block **74**.

Analogous to the electrophilic sulfenylation chemistries utilized in the preparation of thiolated Asp (**61)** and Glu (**64**) building blocks, the synthesis of the suitably protected β-selenol derived Asp (**75**) began with enolization of the side-chain ester of Asp **76** followed by nucleophilic addition to the electrophilic selenium center within the PMB selenosulfonate **77** at a low temperature, which provided the selenylated amino acid **78** in an 81% yield as a mixture of two diastereomers (85:15 *syn:anti,* Scheme 9C). A final deallylation reaction afforded the desired building block **75** as a mixture of *syn* and *anti*-products in excellent yield. A similar synthetic route was also established for the suitably protected β-selenol derived Glu **79**. On this occasion, **79** was prepared as a single stereoisomer (2*S*, 4*S* as determined by NMR spectroscopy) (Mitchell et al., 2017).

### Thiol Derived Tryptophan, Arginine and Glutamine

**SCHEME 10:**
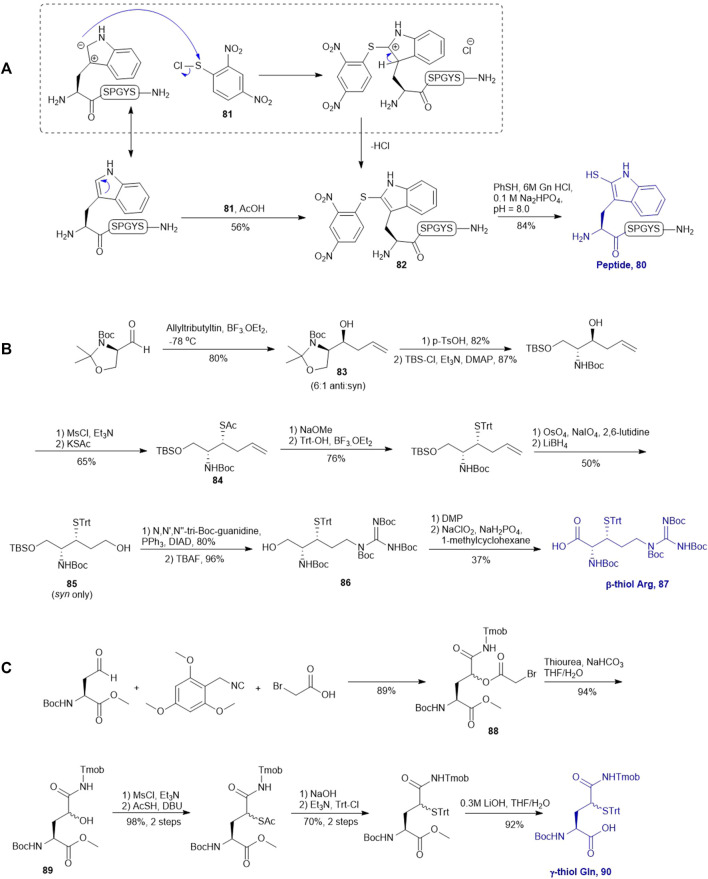
**(A)** Electrophilic sulfeylation of peptidyl Trp; **(B)** Synthesis of β-thiol derived Arg based on Garner’s aldehyde; **(C)** Preparation of γ-thiol derived Gln via a Passerini three-component reaction.

Payne and coworkers also reported an innovative approach to sulfenylate N-terminal tryptophane (Trp) residues in unprotected peptides (Scheme 10A). They first prepared a model peptide bearing an N-terminal Trp residue using Fmoc-based SPPS method. The peptide was then subjected to electrophilic aromatic substitution conditions containing 2,4-dinitrophenylsulfenyl chloride and acetic acid to furnish the Trp-sulfenylated peptide **80** in a 56% isolated yield. The reaction proceeded through electrophilic addition to the 2-position of the indole ring by the sulfenyl chloride **81** followed by hydrogen elimination to re-form the aromatic ring in compound **82** (Malins et al., 2014). This modification can be effectively achieved both on and off resin.

Malins *et al.* described the chemical synthesis of β-thiol Arg based on Garner’s aldehyde (Malins et al., 2013), which included eight high-yielding synthetic steps (Scheme 10B). They first generated an allyl alcohol via nucleophilic addition of allyltributylin to Garner’s aldehyde (Garner, 1984). This reaction proceeded with reasonable stereochemical control, obtaining an inseparable mixture of 6:1 *anti* and *syn* diastereomers (**83**). The *anti*-isomer was generated through a Felkin-Ahn transition state which means that the nucleophile preferentially attacked on the *Re*-configured, less sterically hindered face. The thiazolidine was then deprotected using *p*-toluenesulfonic acid in 1,4-dioxane over 3 h to obtain the free alcohol in a good yield of 82%. After masking the primary alcohol with a TBS moiety and the secondary alcohol was transformed to thioacetate via a mesylation−nucleophilic displacement manifold to provide the *syn*-product **84** in a reasonable yield (62% over 2 steps). The thioacetate protecting group was then converted to an S-Trt protecting group while the allyl group was transformed into an alcohol (**85**) via oxidative cleavage with OsO_4_ and NaIO_4_ followed by reduction with LiBH_4_. At this stage, it was noted that the minor *anti*-isomer formed earlier could be separated from the major product. Next, the *N,N,N*-tri-Boc-guanidine was installed onto **85** via a Mitsunobu reaction to obtain the protected guanidine side chain with a yield of 80%. Next the TBS protected alcohol was deprotected via treatment of tetrabutyl-ammonium fluoride (TBAF) to afford the free alcohol (**86**) in a great yield of 93%. Finally, a two-step sequential oxidation strategy was adopted to afford the desired β-thiol arginine (Arg) **87** in a 37% yield (Malins et al., 2013).

Brik reported the preparation of γ-thiol Gln based on Passerini three-component reaction (Siman et al., 2012) inspired by Kung’s approach on the synthesis of γ-fluorinated Gln (Scheme 10C) (Qu et al., 2011). The side chain of the commercially available Asp was reduced to an aldehyde that was reacted with 2,4,6-trimethoxybenzyl isocyanide and bromoacetic acid to obtain a diastereomer mixture of the γ-acyloxy glutamine (Gln) derivative **88** (*syn*/*anti*, 1:1) in an 89% yield. (Scheme 10C) The bromoacetyl group was then removed using thiourea under basic conditions to give the γ-hydroxyl Gln (**89**), which was subjected to the mesylation-nucleophilic displacement manifold followed by protecting group manipulation to afford the desired γ-thiol derived Gln (**90**) as a mixture of two epimers. This isomeric mixture was used without chromatographic separation for SPPS. Peptide ligation using **90** has been demonstrated on model peptides.

### Photoredox-Catalyzed Asymmetric Giese Reaction

**SCHEME 11:**
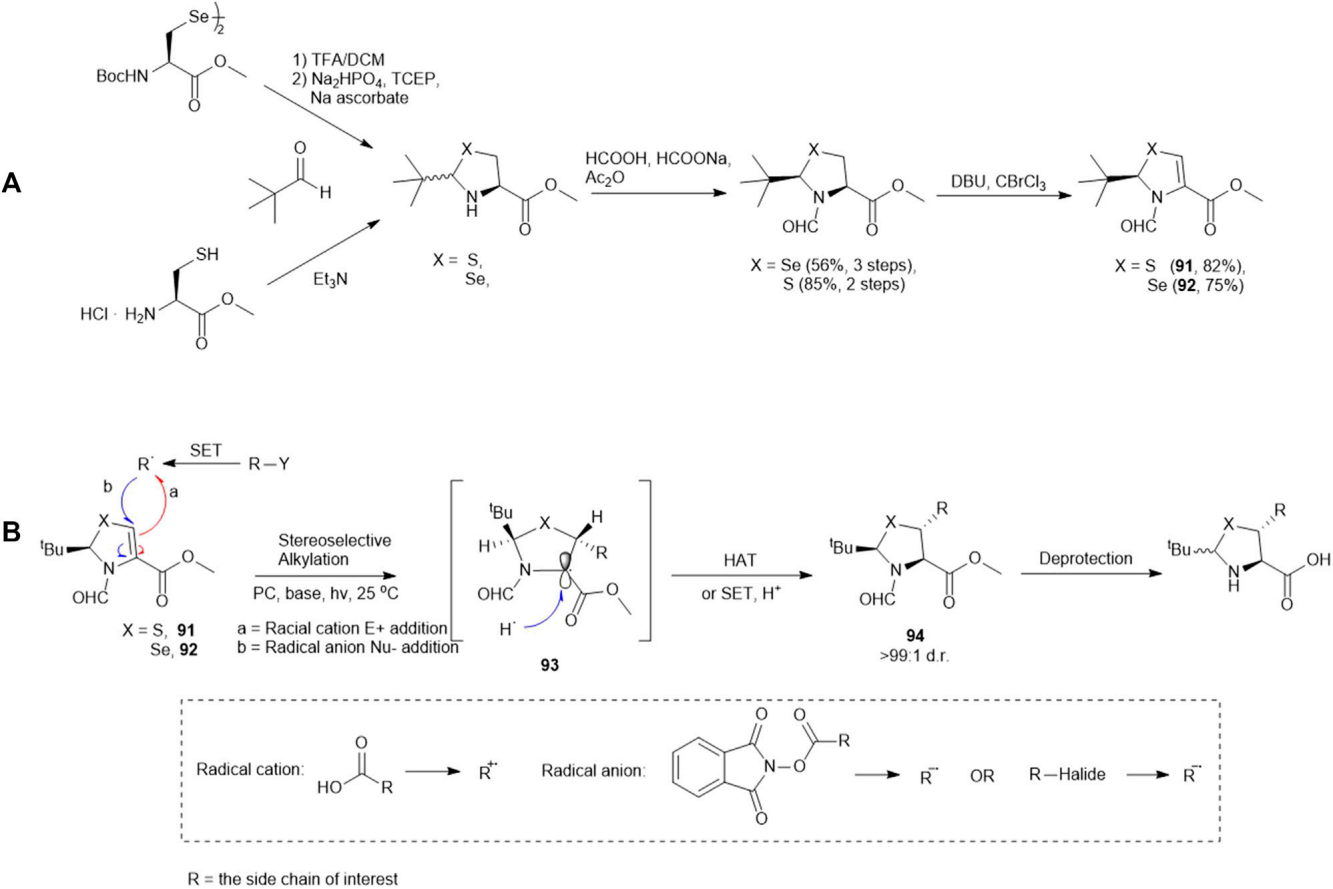
**(A)** Preparation of pivalaldehyde-masked dehydrocysteine and dehydroselenocysteine; **(B)** Wang’s route to β-thiol/selenol derived amino acids via photoredox-catalyzed asymmetric Giese reactions.

Recently, Wang and coworkers have reported the synthesis of a library of enantiopure β-thiolated/selenylated amino acids via a photoredox-catalyzed asymmetric Giese reaction (Yin et al., 2020) (Scheme 11). This approach employed dehydrocysteine and dehydroselenocysteine amino acids protected as thiazoline **91** and selenazoline **92**, respectively. The side chain extension occurred at the β-carbon *via* radical-based 1,4-conjugate addition stimulated by visible light, leading to the formation of a β-thiolated/selenylated amino acid framework. This strategy enabled the preparation of a broad range of β-thiolated/selenylated amino acids on a multigram scale, which included natural and unnatural analogues.

The thiazoline (**91**) and selenazoline (**92**) precursors were easily accessible from the protection of Boc-Sec-OMe and Boc-Cys-OMe with pivalaldehyde followed by hydride elimination to form **91** and **92**. (Scheme 11) The chirality of the auxiliary group determined the stereochemical outcome of the radical addition reaction, whereby the alkyl radical (R^·^) preferred to approach the less hindered *Si*-face of the prochiral β-carbon center. Alkyl radicals can be generated from carboxylic acids (radical cations), redox-active esters (RAEs) or alkyl halides (radical anions) via single-electron transfer (SET) with a suitable photoredox catalyst and light source. The radical intermediate **93** generated in the radical addition was subsequently reduced via hydrogen atom abstraction (HAT) or SET to provide the product **94**. Subsequent removal of the formyl moiety and saponification furnished the desired β-thiolated/selenylated amino acids for SPPS. The *tert*-butylmethyl auxiliary group can be retained to facilitate one-pot, multi-component ligation methods, and removed under acidic conditions after peptide ligation.

Wang’s method represents a generic approach to prepare β-thiolated/selenolated amino acids using Cys and Sec as universal donors. Apart from synthesizing the thiolated/selenylated variants of proteogenic amino acids, the modified forms of unnatural amino acids were also generated, including those with alkyl (primary, secondary and tertiary), pyridine, acetal, ketone and amide substituents on the side chains, suggesting the high structural and chemical tolerance of this method.

## Selected Examples of Using Thiolated and Selenylated Amino Acids in Chemical Synthesis of Post-translationally Modified Peptides and Proteins

### β-Thiol Derived Aspartic Acid

Watson *et al.* reported the implementation of β-thiolated Asp **61** in the total synthesis of post-translationally sulfated anopheline proteins derived from *Anopheles* mosquitos (Watson et al., 2018). Selected sulfoproteins from *A. gambiae* and *A. albimanus* were first expressed in *Trichoplusia ni* insect cells and directed to the secretory pathway by a honeybee mellitin leader sequence. The secretory anopheline proteins in cell medium were submitted to tandem mass spectrometry analysis to reveal the tyrosine (Tyr) sulfation sites (Watson et al., 2018). They next sought to systematically characterize the anticoagulant activity of individual sulfation forms of each anopheline protein and identify the most potent sulfated form for therapeutic development. To this end, they initially attempted to integrate amber-stop codon suppression technology with a cell-free expression system to generate anopheline proteins with amber-codon incorporation of Tyr sulfates. However, this synthetic pipeline was unable to produce the doubly sulfated *A. albimanus* protein and cannot yield sulfoproteins in sufficient quantity for detailed *in vitro* and *in vivo* characterization. Therefore, it was decided to chemically synthesize the sulfoprotein libraries of anopheline proteins from *A. albuimanus and A. gambiae* under a ligation-desulfurization manifold (Watson et al., 2018).

**SCHEME 12:**
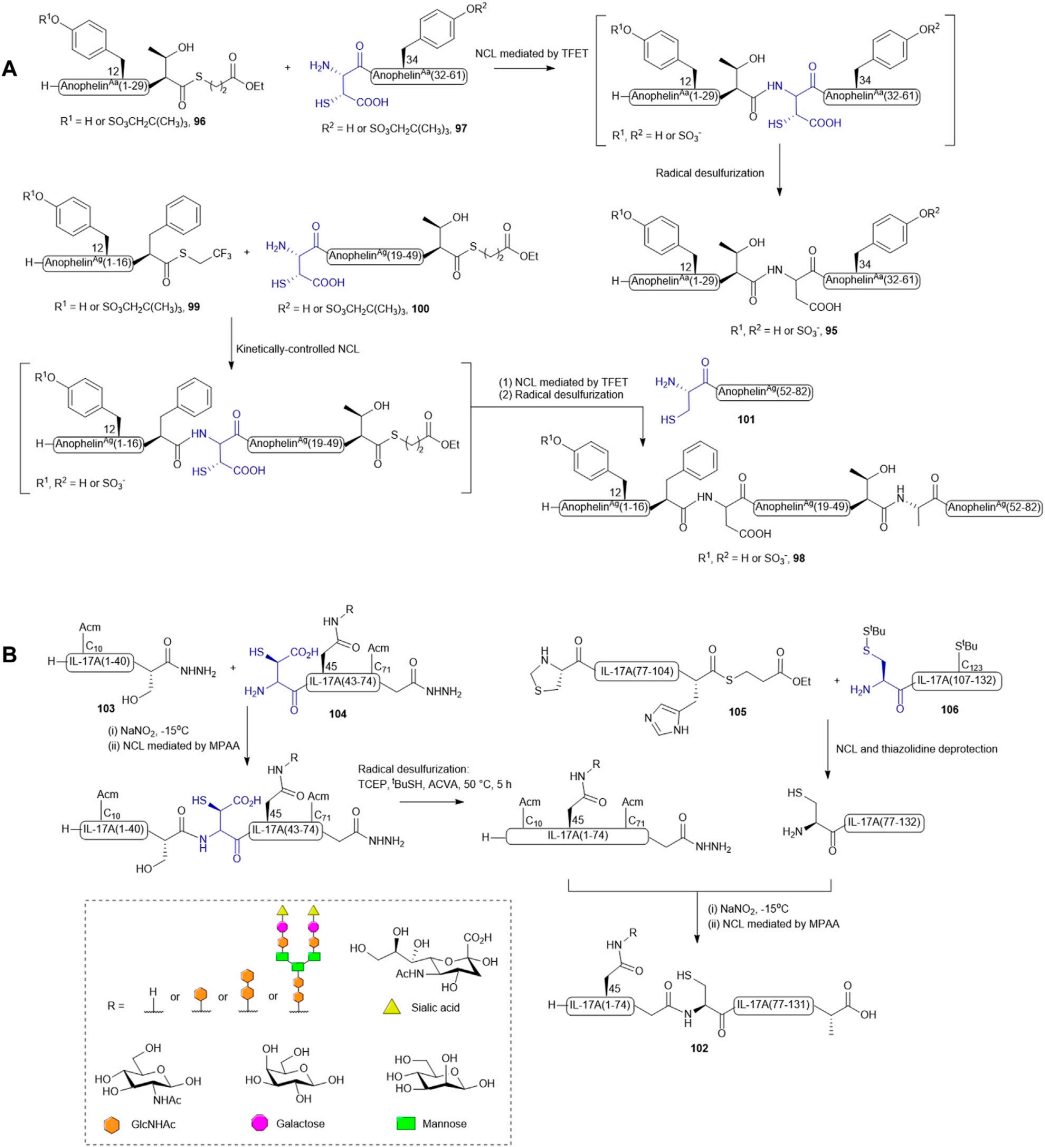
**(A)** Synthesis of sulfated anophelin variants via one-pot ligation-desulfurization chemistry; **(B)** Convergent synthesis of homogeneous glycoforms of IL-17A.

As natural anopheline proteins do not possess a cysteine residue that enables peptide assembly through NCL, the retrosynthetic strategy was therefore designed based on ligation−desulfurization methodology (Scheme 12A). *A. albimanus* protein **95** was disconnected at the center between Thr30 and Asp31, resulting in two fragments: the N-terminal peptide thioester fragment (**96**) containing either sulfated or unsulfated Tyr12 and a C-terminal fragment (**97**) composed of a β-thiol Asp residue as ligation handle and a sulfated or unsulfated Tyr34 (Watson et al., 2018). These fragments were ligated in aqueous denaturing buffer aided by trifluoethanethiol (Thompson et al., 2014). The ligation reactions proceeded to completion within 16 h followed by radical desulfurization chemistry mediated by VA-044, TCEP and reduced glutathione, which ultimately afforded the desired proteins in good yields. A sequential ligation−desulfurization methodology was implemented in the synthesis of sulfated *A. gambiae* protein **98** (Watson et al., 2018). The authors produced three suitably functionalized peptide fragments via Fmoc-based SPPS for convergent assembly of the desired protein: the N-terminal fragment **99** containing a C-terminal TFET thioester with the desired sulfated Tyr12 protected by a neopentyl group, the middle fragment (**100**) possessing an N-terminal β-thiol-derived Asp residue and a C-terminal deactivated alkyl thioester, and the last fragment (**101**) containing a cysteine residue as a ligation handle in place of the native Ala residue (Watson et al., 2018). The sulfoprotein was synthesized in a one-pot manner beginning with kinetically-controlled, sequential assembly of the three fragments from an N-to-C direction followed by radical desulfurization chemistry to eliminate the thiol appendages. All sulfated anopheline proteins prepared through this approach exhibited higher inhibitory activities against thrombin *in vitro* and *in vivo* compared to unmodified counterparts (Watson et al., 2018).

Recently, β-thiolated Asp has also been used for the design and synthesis of a homogenously N-glycosylated interleukin-17A (IL-17A) with the aim to elucidate the impacts of N-glycan on protein folding, thermal stability, and inflammatory cytokine inducing ability (Li et al., 2021b). In particular, the ligation-desulfurization methodology provided a powerful means to access individual glycosylated forms of IL-17A that are otherwise difficult to recombinantly derive. IL-17A (**102**) was disconnected into four peptide segments prepared through Fmoc-based SPPS method, and methionine (Met) residues were mutated to norleucines to prevent unwanted oxidation. Both segments (1-41) **103** and (42-75) **104** contained a C-terminal peptide hydrazine which serves as a latent thioester, whilst segment (76-105) **105** was modified with a C-terminal alkyl thioester (Scheme 12B) (Fang et al., 2011; Li et al., 2021b). A β-thiol-derived Asp was introduced to the N-terminus of segment (42-75) **104**, which permits a NCL reaction to form the first half of IL-17A (1–75), whereas two orthogonally protected cysteines were adopted in preparing the N-terminal residues of segments (76-105) **105** and (106–132) **106**. The peptide assembly of each glycosylated form of IL-17A began with performing NCL between segments (75-105) **105** and (106–132) **106** and peptide hydrazine ligation between segments (1-41) **103** and (42-75) **104** in parallel to furnish the first and second halves of IL-17A. Unmasking of the thiazolidine-protected Cys76 enabled the final convergent assembly of the two main fragments (1–75) and (76-105) to provide the full-length IL-17A **102** (Li et al., 2021b).

This convergent approach provided plasticity in the synthesis of discrete glycosylated forms of IL-17A since each segment could be modified and optimized independently to maximize the synthetic yield and PTM diversity. For peptide **104** derived with N-acetyl glucosamine (GlcNAc) or a disaccharide [GlcNAc(β1→4)GlcNAc], the respective N-glycosylated Asn amino acids were incorporated as Fmoc-protected building blocks in SPPS. However, to obtain the glycoform of **104** bearing a complex biantennary sialyloligosaccharide, the N-glycan chain was developed on the GlcNAc moiety within the target peptide using the Endo-M-catalyzed transglycosylation method. The N-terminal thio-aspartyl residue in the glycopeptide can still mediate the ligation reaction smoothly without an observable impact on the integrity of the glycan. However, an inseparable by-product containing a VA-044 adduct on the β-thiolated Asp residue was formed in the radical desulfurization reaction using Danishefsky’s conditions. The authors postulated that the carboxylate side chain of the thioaspartyl residue forms a tight ion pair with the basic 4,5-dihydroimidazole radical generated from homolytic cleavage of VA-044, leading to competitive interception of the radical propagation chain mediated by sulfhydryl donors and TCEP. To their delight, using a different radical initiator ACVA possessing electronegative carboxylates that could repel the binding of Asp significantly diminished this side reaction and allowed completion of the total synthesis. The resulting homogeneous glycoproteins have enabled detailed structure-based evaluation of protein stability, proinflammatory activities, and receptor binding abilities (Li et al., 2021b).

### β-Thiol Derived Arginine

**SCHEME 13:**
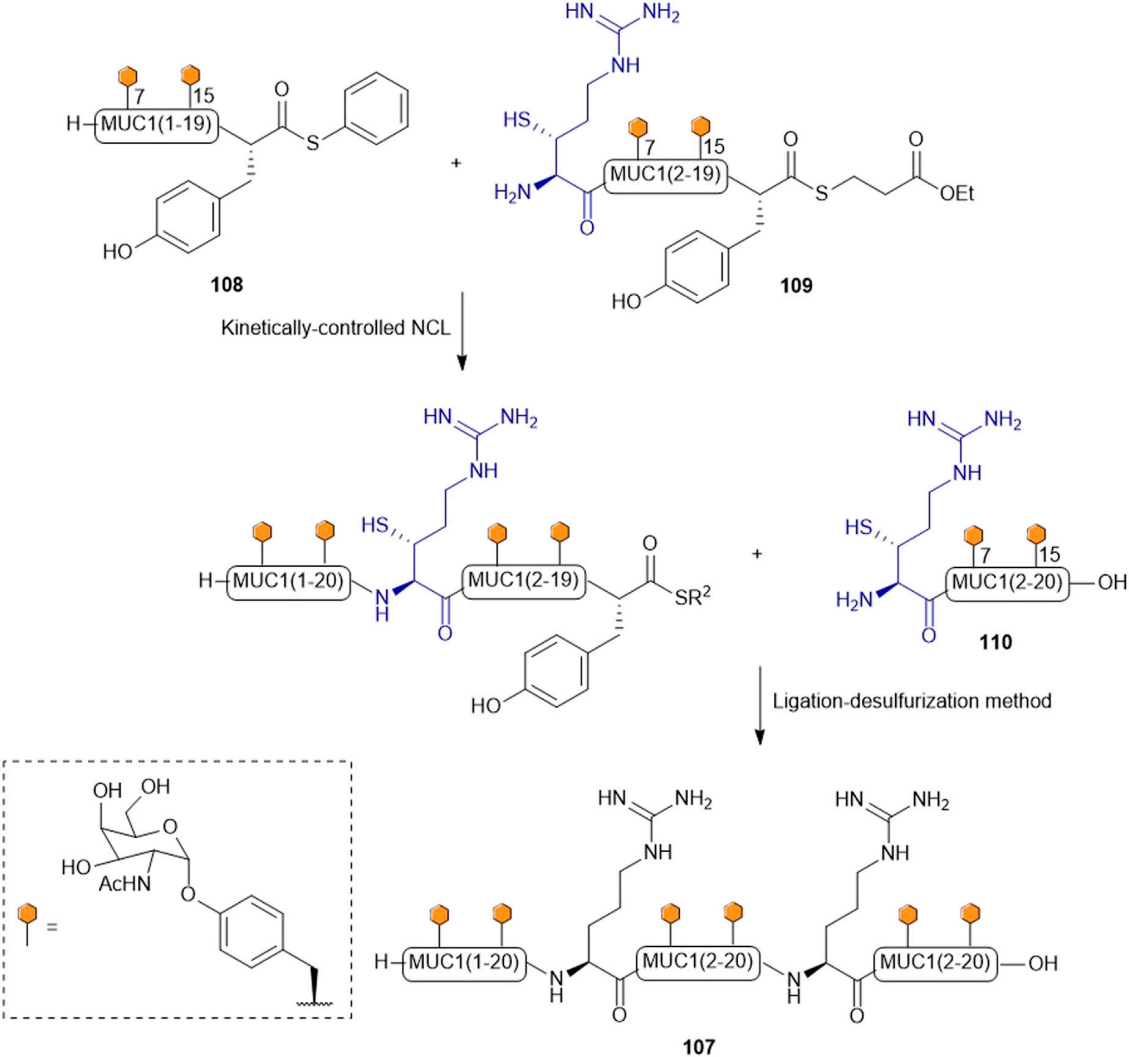
Synthesis of MUC I glycopeptide using a ligation-desulfurization method.

Payne and coworkers highlighted the effectiveness of their β-thiol Arg amino acid in NCL chemistry by designing and synthesizing the glycopeptide (**107**) of the extracellular domain of mucin 1 (MUC1) (Scheme 13) (Malins et al., 2013). They employed a one-pot kinetically-controlled ligation method to assemble the target of interest, where fragment one (**108**) was a glycopeptide bearing an activated phenyl thioester, the middle glycopeptide fragment contained an N-terminal β-thiol Arg and a C-terminal alkyl thioester (**109**), and the last fragment also possessed an N-terminal β-thiol Arg as the ligation handle (**110**). Taking full advantage of the high reactivity of phenyl thioesters in NCL, the intermolecular ligation reaction between **108** and **109** proceeded with much faster kinetics than intramolecular cyclization of **109**. Upon completion, the final fragment (**110**) was added along with 2 vol% thiophenol to activate the final ligation, which went to completion in 31 h to produce the glycopeptide (**107**) in 43% yield after purification. This peptide was then desulfurized to afford the native glycopeptide in 38% isolated yield (Malins et al., 2013).

### δ-Thiol Derived Lysine

**SCHEME 14:**
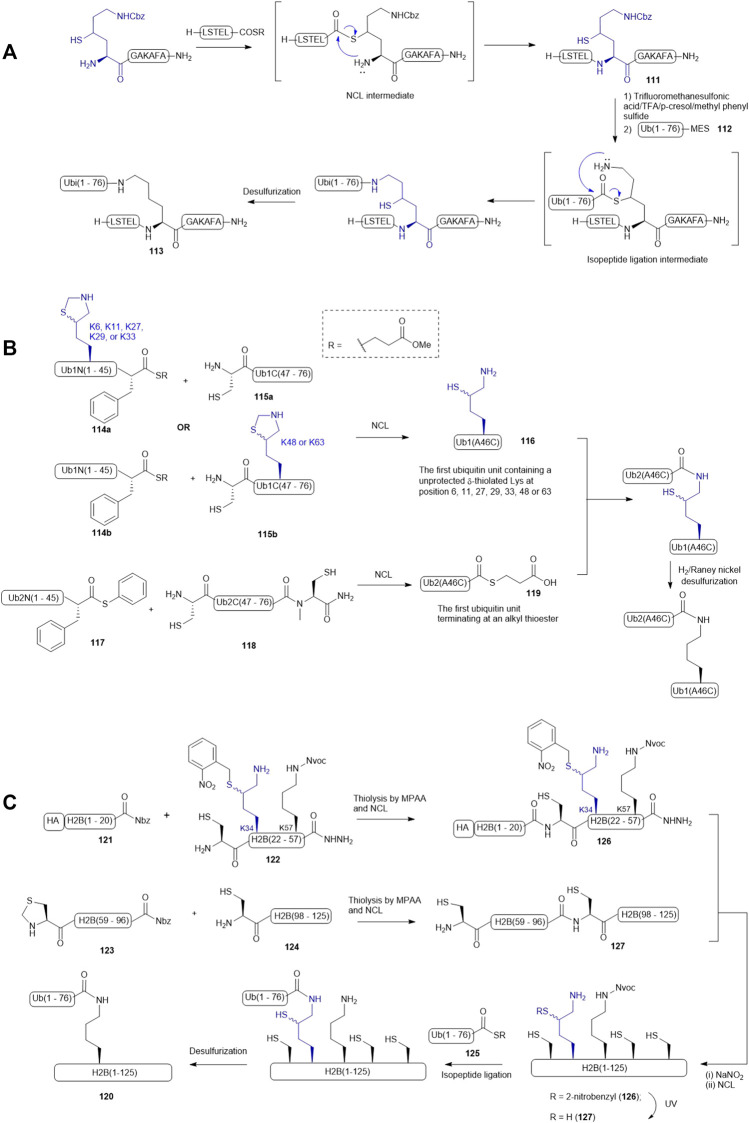
**(A)** Liu’s dual ligation strategy to access site-specific ubiquitination; **(B)** Brik’s synthesis of the di-ubiquitin library **(C)** Siman’s route to access Lys34-ubiquitinated H2B.

Liu and coworkers demonstrated the use of γ-thiol derived Lys in a dual peptide ligation methodology that enabled streamlined synthesis of ubiquitinated peptides and proteins (Yang et al., 2009). For example, a γ-thiol derived Lys having a Cbz-protected side chain was introduced to the N-terminus of a model peptide (**111**), allowing for the construction of the primary sequence of the target peptide (**111**) via NCL (Scheme 14A). The Cbz protecting group was then removed using a chilled cocktail of TFMSA/TFA/p-cresol/methyl phenyl sulfide (1:7:1:1, v/v/v/v). After HPLC purification, isopeptide ligation was performed by adding a ubiquitin mercaptoethanesulfonate (MES) thioester **112**. The reaction was complete within 45 min followed by desulfurization to furnish the ubiquitinated peptide **113** in excellent yield (90%).

δ-thiolated Lys has also been extensively utilized by other research groups for site-specific incorporation of a ubiquitin protein. Brik and coworkers published a synthetic route to all possible di-ubiquitin variants linked through disparate Lys-isopeptide bonds (Scheme 14B) (Kumar et al., 2010). The first ubiquitin unit within the dimer construct was disconnected at the Phe-Ala junction to afford two peptide segments (**114** and **115**): the N-terminal fragment **114** contained a phenylalaninyl MES thioester, whereas an unprotected Cys residue was integrated as the N-terminal residue of fragment **115**, and a δ-thiol Lys residue was introduced to either peptide segment according to the isopeptide linkage of preference. Each ligation was performed under standard NCL conditions, proceeding to completion after 7 h. This was followed by *in situ* deprotection of the side chain of the δ-thiol Lys residue to afford the desired ubiquitin unit (**116**) with a thiol-derived Lys residue at a designated position. The second ubiquitin unit was again divided at the Phe-Ala junction: the N-terminal segment was derived with a phenylalanyl thioester (**117**), whereas the C-terminal segment (**118**) was equipped with a ligation handle and a latent thioester (N-methylcysteine). After the ligation reaction, the crude product was incubated with 20% mercaptopropionic acid for 16 h at 40^o^C to afford the desired ubiquitin thioester **119** for final ligation. To assemble all the di-ubiquitin variants, seven peptide ligation reactions were carried out in parallel. Plausibly due to the large protein size, each reaction required more than 24 h to afford a moderate isolated yield (35–40%). The authors also noticed that not all the thiol auxiliaries could be removed under radical desulfurization conditions and thus, H_2_/Raney nickel conditions in combination with TCEP treatment were adopted to desulfurize the di-ubiquitin variants.

Chromatin packaging and function are tightly regulated by histone ubiquitination modifications, however, the biological roles of site-specific ubiquitin modifications of H2B remained unclear, prompting Siman *et al.* to chemically synthesize H2B homologues (**120**) with defined ubiquitination patterns (Siman et al., 2013). Since H2B lacks a native Cys residue, they adopted a ligation−desulfurization strategy wherein Ala21, Ala58, and Ala97 were mutated to Cys residues for NCL purposes, meaning the H2B protein would be divided into four segments, including H2B(1–20) **121** with an HA tag, H2B(21–57) **122**, H2B(58–96) **123**, and H2B(97–125) **124**. Initially, the synthesis started with ligating a ubiquitin thioester (**125**) to the side-chain of the δ-thiolated Lys at position 34 while the N-terminal Cys residue of H2B (21–57) was masked by an acetamidomethyl (Acm) group. The Acm group was then removed to allow for ligation with the H2B(1–20) thioester **121**. However, the resultant ligation product H2B(1–57) did not survive in the conditions employed to activate acyl hydrazide. The synthetic strategy was therefore altered and focused on completing the synthesis of the full-length H2B protein before isopeptide ligation with the ubiquitin thioester **125**. Hence, the H2B(21–57) was re-synthesized by introducing a 2-nitrobenzyl (NB) group to mask the δ-thiol derived Lys34 (peptide **122** in Scheme 14C). The ligation of peptides **121** with **122** proceeded to completion in a good isolated yield; the following oxidation and thioesterification of the ligation product also proceeded in a straightforward manner to form **126**. It was subjected to NCL conditions containing the ligation product (**127**) of **123** and **124** to provide the full-length H2B (**128**) bearing a photo-caged thiolated Lys at position 34. After removing the NB group under UV irradiation, the unmasked thiolated H2B **129** was ligated to the ubiquitin thioester **125** and then subjected to desulfurization conditions to afford the desired Lys34-ubiquitinated H2B protein **120**. The authors noticed a significant amount of Met oxidation products were formed in the photo-deprotection and isopeptide ligation conditions and suggested norleucine would overcome this issue in future studies.

### β-Selenol Derived Phenylalanine

**SCHEME 15:**
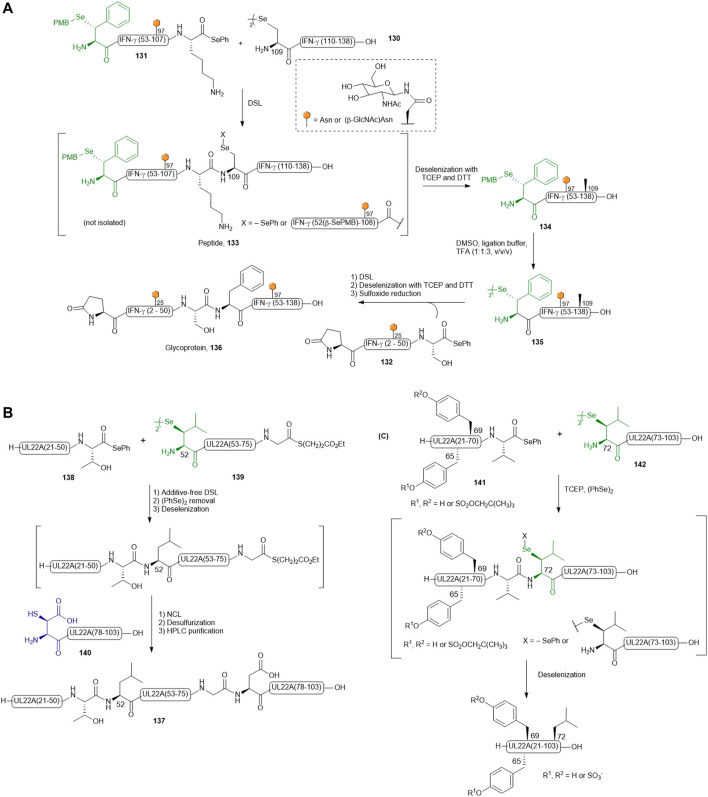
**(A)** Payne’s route to access differentially glycosylated human IFN-γ; **(B)** Chemical synthesis of post-translationally sulfated UL22A via β-selenylated Leu-mediated DSL and **(C)** reductive diselenide-selenoester ligation.

Recently, Payne and coworkers reported the preparation of four discrete glycosylated forms of human IFN-γ using a tandem DSL−deselenization strategy (Wang et al., 2020a). Specifically, the human IFN-γ sequence was disconnected at Ser51−Phe52 and Lys108−Ala109 junctions, which generated three classes of differentially functionalized fragments to synthesize (Dawson et al., 1994): a C-terminal fragment, IFN-γ(109–138) **130**, where the N-terminal Ala was replaced with a Sec residue (Bang et al., 2006), a highly functionalized middle fragment [IFN-γ(52–108), **131**] having a PMB-protected β-selenol Phe at the N-terminus and a phenylselenoester at the C-terminus, with/without a β-GlcNAc moiety on the side chain of Asn97, and (Thompson et al., 2014) the N-terminal fragment **132** of IFN-γ(1–51) possessing an N-terminal pyroglutamate and a C-terminal phenylselenoester with either unmodified or β-GlcNAc-derivatized Asn25. The first DSL reaction was performed by simply mixing the diselenide dimer **130** of IFN-γ (109–138) bearing an N-terminal selenocystine (1.0 equiv.) with the middle fragment of IFN-γ(52–108) (**131**, 1.1 equiv.) in ligation buffer under additive-free conditions. After completion within 5 min, the desired product was composed of a mixture of the branched selenoester product and asymmetric diselenide products (termed peptide **133**). The resulting ligation mixture was subjected to deselenization conditions immediately, which involved removing diphenyl diselenide through hexane extraction followed by the addition of the deselenization buffer containing TCEP (50 equiv.) and DTT (50 equiv.). After 16 h, purification by HPLC afforded the PMB-protected IFN-γ (52–138) **134** in good isolated yields (62 and 57%, respectively). The β-selenol phenylalanine residues of **134** were subsequently unmasked under oxidative deprotection conditions comprising DMSO, ligation buffer (6 M Gdn HCl and 0.1 M Na_2_HPO_4_) and TFA in a ratio of 1:1:3 (v/v/v) to generate the desired peptide diselenide dimer **135** (81–90%). The deprotection conditions also caused concomitant oxidation of the Met residues. The final DSL reaction to assemble hIFN-γ (52–138) and (1–51) fragments was performed by dissolving **135** and hIFN-γ(1–51) selenoester **132** with/without β-GlcNAc-modified N25 residue in additive-free ligation buffer at a higher dilution (1–2 mM on the basis of the monomeric form of **132**). The ligation reaction proceeded smoothly within 30 min and was subjected to deselenization conditions to afford the four homogeneously glycoforms of hIFN-γ(1–138) in excellent yields (54–59% over two steps). Finally, the oxidized Met residues were reduced under Hackenberger’s conditions to afford four glycosylated variants of IFN-γ **(**termed glycoprotein **136** in Scheme 15A) (Hackenberger, 2006).

### β-Selenol Derived Leucine

To expand the scope of one-pot DSL−deselenization methodologies, the utility of β-selenyl Leu was highlighted in the synthesis of a chemokine-binding protein from human cytomegalovirus (UL22A) (Wang et al., 2017) by Wang *et al.* UL22A has been shown to neutralize RANTES effectively *in vitro* and was demonstrated by the authors that the Tyr residues 65 and 69 were post-translationally sulfated, leading to further enhancement in chemokine binding. Taking advantage of the distinct reaction kinetics of DSL and NCL, a one-pot three-fragment ligation starting with DSL (using β-Se-Leu) followed by NCL (mediated by β-thiol-derived Asp) was planned (see Scheme 15B) (Wang et al., 2020b). As such, the full-length protein **137** was divided into the three fragments, including an N-terminal segment UL22A 21–51 (**138**) bearing a phenyl selenoester at its C-terminus, a bifunctional middle fragment (UL22A 52–76, **139**) having a C-terminal alkyl thioester and an N-terminal β-selenyl Leu as diselenide dimer, and the last fragment UL22A (77-103) **140** possessing an N-terminal β-thiolated Asp. The phenyl selenoester **138** was first conjugated to the middle fragment dimer **139** following a standard DSL protocol, which proceeded to completion within 30 min. The fast ligation kinetics of DSL in the absence of a thiol additive meant that no intramolecular cyclization of **139** was possible. The C-terminal fragment UL22A (77-103) **140** was subsequently added together with TCEP (25 mM) and TFET (2 vol%) to effect the final ligation with concomitant deselenization. This was followed by radical desulfurization *in situ* to convert β-SH-Asp into native Asp, providing UL22A (21–103) **137** in 40% yield over 4 steps after HPLC purification.

In order to improve synthetic efficiency, a two-fragment ligation strategy was also employed to synthesize UL22A, where the protein was disconnected at the Val71-Leu72 junction to provide fragments **141** and **142** (Scheme 15C) (Wang et al., 2017). Interestingly, the DSL reaction did not proceed significantly, attributed to the steric congestion at this Val-Leu junction. The addition of diphenyl diselenide and TCEP enabled reduction of the diselenide dimer and unveiling the nucleophilicity of the selenolate allowed efficient convergent assembly within 1 h. The need for additives to facilitate ligation at the Val71-Leu72 junction but not in the DSL reaction at the Thr51-Leu52 junction highlighted the fact that DSL reactions are dependent on the specific nature of the fragments, not only on the steric hindrance around the selenoester center.

### γ-Selenol Derived Proline

**SCHEME 16:**
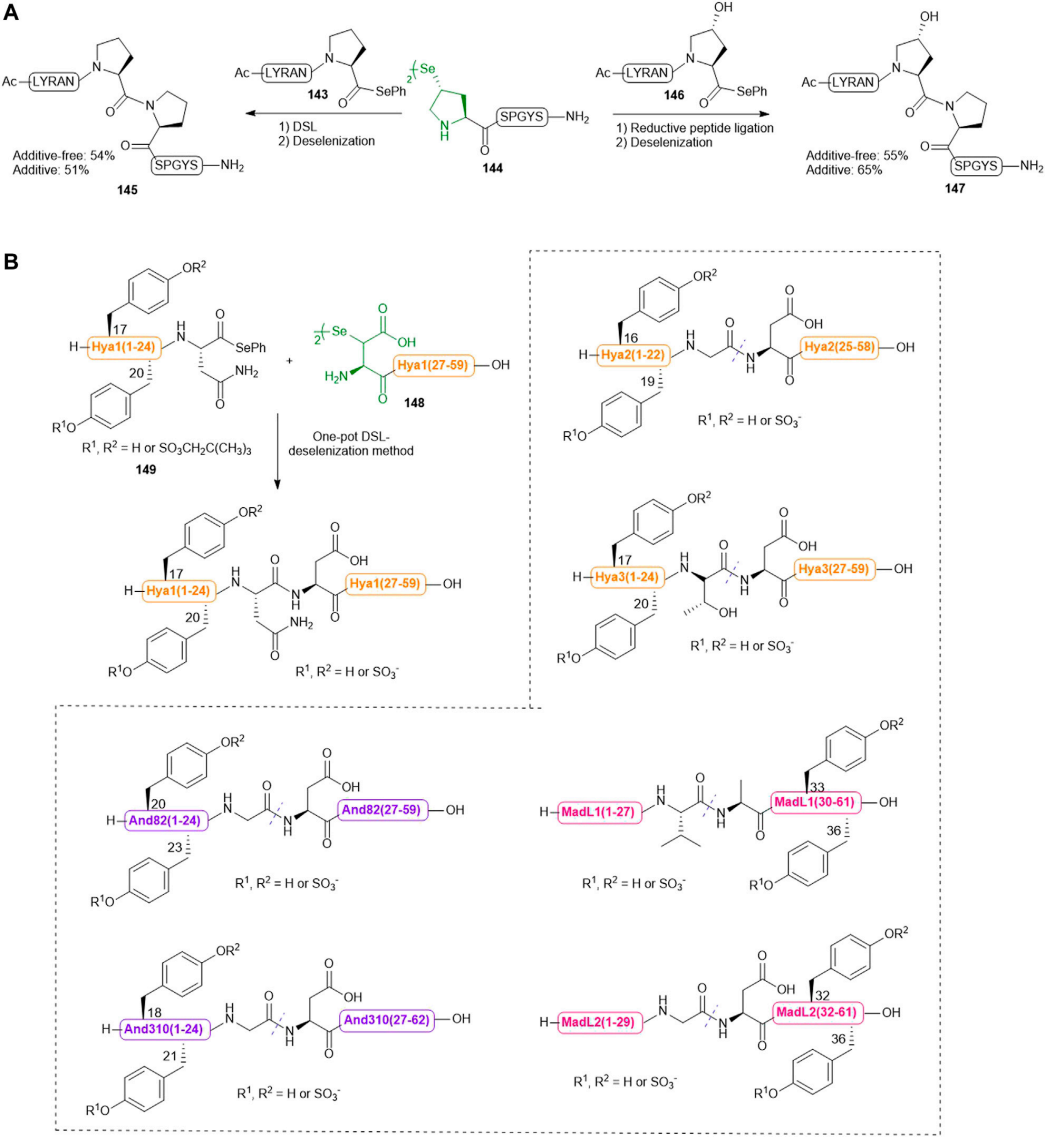
**(A)** Pro-Pro ligation via DSL and reductive selenium ligation chemistries; **(B)** Chemical synthesis of a library of sulfated anticoagulants via a DSL-deselenization manifold.

Recently, Sayers *et al.* harnessed the complementary reactivities of *trans*-γ-selenyl Pro and prolyl selenoesters to facilitate DSL reactions at the notorious proline−proline ligation junction for the first time (Scheme 16A) (Sayers et al., 2018a). The authors first demonstrated the capacity of γ-selenyl Pro in mediating DSL chemistry (within 5–45 min) using peptide selenoesters terminated at different C-terminal residues. After this initial assessment, the authors investigated whether this selenylated Pro warhead could effect DSL at intractable Pro−Pro junctions. Excitingly, the ligation reaction between a model peptide Ac-LYRANP-SePh (**143**) (2.0 equiv.) and the peptide diselenide **144** (1.0 equiv. with respect to the monomer) proceeded to completion to form **145** in 16 h in 6 M GnHCl/0.1 M phosphate buffer at pH 6.2. Upon completion, the ligation mixture was extracted with hexane to remove the diphenyl diselenide precipitate followed by *in situ* deselenization with TCEP and DTT to provide the desired peptide in a good yield. The authors also demonstrated the utility of this ligation chemistry in chemical synthesis of peptides bearing hydroxyPro-Pro junctions. They additionally examined whether the inclusion of TCEP could accelerate the Pro-Pro ligation reaction. To prevent early deselenization by TCEP (50 mM), diphenyl diselenide (20 mM) was also added as a radical trap. The reaction between peptide diselenide **144** and selenoesters **143** and **146** proceeded to completion smoothly under reductive ligation conditions, leading to high-yielding synthesis of **144** and **147** after deselenization and HPLC purification. However, there was no significant improvement in the ligation rate compared to that of DSL.

The enabling nature of such Pro-Pro ligation-deselenization methods was also demonstrated by the high-yielding chemical synthesis of the submaxillary gland androgen regulated protein 3B and lumbricin-1, which represented the first two successful examples of chemical protein synthesis through joining the sterically and electronically demanding Pro-Pro junctions.

### β-Selenol Derived Aspartic Acid

Previously, Payne and coworkers demonstrated that hematophagous ticks and mosquitos produce a suite of Tyr-sulfated protein anticoagulants which hijack the host’s central clotting machineries to facilitate blood ingestion (Thompson et al., 2017; Watson et al., 2018). Recently, they utilized a bioinformatic approach to identify multiple putative thrombin inhibitors [hyalomin (Hya) 1, 2 and 3, andersonin (And) 82 and 310, and madanin-like protein (MDL) 1 and 2] and their two conserved Tyr sulfation sites. To confirm the biological activity of each sulfated Tyr residue and investigate the functional interplay between the PTM and the surrounding amino acids on anticoagulant mechanism, the sulfated variants of these anticoagulants were synthesized via a one-pot DSL−deselenization manifold (Scheme 16B) (Watson et al., 2019).

The requisite peptide selenoesters and their peptide diselenide companions were prepared by Fmoc-based SPPS method. In particular, the sulfated Tyr residues were masked by neopentyl groups and incorporated into the selenoester segments of Hya1, Hya2, Hya3, And82 and And310 proteins, or the peptide diselenide segments of MadL1 and MadL2. β-selenyl Asp amino acid was introduced to the N-terminus of each peptide diselenide segment (i.e., the Hya1 (27–59) fragment **148** shown in Scheme 16B) except that of MadL1, wherein selenocystine was incorporated instead. All proposed anticoagulants were assembled via DSL chemistry, whereby each peptide selenoester (i.e., the Hya1 (1–24) fragment **149** in Scheme 16B) and the ligation partner were first prepared as a 10 mM solution (with respect to the monomer) in ligation buffer (6 M Gn. HCl, 0.1 M Na_2_HPO_4_, pH 6.0–6.5) separately, followed by mixing of the two solutions in an equal volume to effect the ligation reaction. 10 vol% N,N-dimethylformamide was also added to the DSL mixture of Hyd and fragments in order to facilitate peptide dissolution. All reactions proceeded to completion within 20 min, which were subsequently subjected to deselenization conditions containing the final concentrations of 250 mM TCEP and 25 mM DTT. In contrast to the long reaction time (16 h) required for converting selenocystine to Ala residues, deselenization at the β-selenyl Asp residue was complete within 10 min owing to resonance stabilization of the β-carbon–centered radical. After deselenization, the crude mixtures were further incubated for 8–16 h to allow complete removal of neopentyl protecting groups before purification by reversed-phase HPLC, which afforded the target library of homogeneous sulfated variants of Hya, And and MDL in excellent yields.

### Strategic Considerations in Chemical Protein Synthesis

NCL was developed to overcome the size limitation of synthetic polypeptides imposed by solid-phase peptide synthesis (SPPS), enabling one to convergently build larger polypeptides and proteins from smaller peptide fragments. However, the efficiency of the SPPS processes undertaken to prepare the requisite fragments is still a critical factor to consider, which may shape the retrosynthetic design. Typically, SPPS can afford polypeptides having 40–50 residues in length, but preparing shorter peptide segments may be required for difficult sequences and for those which do not involve turn-inducing elements (i.e., Pro and pseudoproline residues). In general, dividing the protein target into 30–40 residue fragments provides a good chance to obtain the requisite ligation segments in reasonable yields and adequate purity (>95%). Another crucial factor to consider is the chemical reactivity of the amino acids within the ligation junctions and their potential influence on the processes of ensuing chemistries. Conventionally, retrosynthetic disconnections were decided based upon the location of native Cys residues within a target protein, but the development of thiol- and selenol-derived amino acids has massively expanded our choices. Careful consideration of the chemical orthogonality of these modified amino acids may allow one to develop streamlined synthesis of the target protein harnessing a one-pot, multicomponent ligation method or *in situ* desulfurization/deselenization method with a view to improve the overall synthetic efficiency and minimize laborious work of intermediary purification. For example, Thompson *et al.* reported a chemoselective desulfurization method to remove the β-thiol auxiliary at Asp residues in the presence of free Cys residues using TCEP and dithiothreitol (DTT) at pH 3, which makes β-mercapto Asp attractive in modern total synthesis of proteins possessing native Cys residues (Thompson et al., 2013). The influence of the thiolated/selenylated chiral centers on ligation efficiency should also be considered. For instance, Danishefsky and co-workers demonstrated that the ligation rate of the *erythro* isomer of β-thiol Leu is prohibitively slow while the *threo* form allows ligation with Gly, Ala, Phe and Val thioesters to complete within 8 h (Tan et al., 2010). Likewise, it has also been shown that the *trans* isomer of γ-thiol Pro can mediate fast NCL reactions comparable to other thiolated amino acids, whereas the *syn* isomer prevents S to N acyl shift during the process of NCL (Ding et al., 2011; Townsend et al., 2012).

The rate of NCL is also strongly influenced by the steric and electronic factors of the C-terminal amino acid residue that acts as an acyl donor. For example, thioester-derived β-branched amino acids (i.e., Val, Ile and Thr) react sluggishly with the ligation counterpart leading to the accumulation of by-products arising from the hydrolysis and other side reaction pathways (Hackeng et al., 1999; Siman et al., 2012). Prolyl thioesters were found to be poor acyl donors due to the n→π* electronic interaction between the carbonyl group on the Pro nitrogen to the thioester carbonyl carbon resulting in a reduction in electrophilicity (Hackeng et al., 1999; Pollock and Kent, 2011). Durek and Alewood provided a simple solution to these issues by introducing alkyl/aryl selenoesters to the C-terminal residues, which resulted in orders of magnitude enhancement of ligation rates, as exemplified by the model ligation reaction with a prolyl selenoester proceeding to completion within 2 h (Durek and Alewood, 2011). Finally, peptide thioesters terminating at a C-terminal Asp, Glu, Asn, Gln or Lys residue, are prone to cyclize *via* nucleophilic attack at the acyl center by the C-terminal side-chain functionality. The cyclization reaction at Lys, Asn and Gln residues may be suppressed by performing the ligation at pH 6–7; however, it is challenging to prevent the cyclization at Asp and Glu residues without masking their carboxylate side chains (Barnes et al., 2021).

It is also important to note the disparate reactivity profiles of thiolated amino acids in desulfurization chemistries, which may allow one to design a more strategic approach to remove the thiol auxiliaries. Penicillamine as a precursor of Val was commonly used in developing ligation–desulfurization strategies due to its commercial availability (Chen et al., 2008; Haase et al., 2008; Reimann et al., 2015). Interestingly, Haase *et al.* reported that desulfurization yields were moderate to low when using metal-based methods whilst Danishefsky’s radical approach could afford quantitative conversion to the Val residue (Haase et al., 2008). β-mercapto Asp and γ-mercapto Glu were commonly removed via the desulfurization chemistry initiated by VA-044 (Thompson et al., 2014); however, certain peptide sequences can promote a side reaction between the VA-044 radical and the sulfanyl radical generated via hydrogen abstraction from the sulfhydryl auxiliary (Cergol et al., 2014; Li et al., 2021b). An alternative radical initiator, 4,4′-azobis (4-cyanovaleric acid) reported by Li *et al.* was shown to effectively suppress this deleterious side reaction and facilitate conversion to the desired product (Li et al., 2021b). Brik and coworkers reported that ligation products containing γ-mercapto Gln were decomposed to unidentified compounds in metal-free desulfurization conditions due to the unstable nature of the γ-thiolated side chain (Siman et al., 2012), whereas clean conversion to the final product could be achieved in the nickel boride conditions. Likewise, Malins *et al.* reported that the sulfanyl moiety in 2-thiol Trp could not be eliminated in radical-based conditions plausibly due to the strength of the C–S bond and the contributions of the other tautomeric form, but a metal-based reductive cleavage protocol with hydrogen gas and Pd on Al_2_O_3_ was applied to facilitate the formation of the desulfurized product without detectable demethylthiolation on Met residues (Malins et al., 2014).

Despite being the chalcogenic relative of thiol, selenols manifest markedly different chemical reactivity to thiols. It is well documented that selenol auxiliaries possess a higher acidity and greater nucleophilicity than thiols and can be readily removed in the presence of other proteogenic functionalities (using TCEP and DTT at pH 6–7) (Gieselman et al., 2001; Metanis et al., 2010). The ease and practicality of deselenization chemistry have attracted enormous interest in expanding the scope of selenol-derived amino acids in modern total synthesis of proteins. Due to the thermodynamically favourable reaction between TCEP and selenol leading to homolytic cleavage of the C−Se bond, aryl thiols were often used as alternative reductants in selenol-assisted NCL. However, the weak reducing power of aryl thiols only affords a low level of productive selenol during ligation; hence, the rate of ligation does not benefit from the greater reactivity of selenol. Metanis and coworkers demonstrated that the deleterious reaction between Sec and TCEP can be suppressed by the addition of ascorbate as a radical scavenger, and the resulting additive combination allows one to fully exploit the latent reactivity of selenol (Reddy et al., 2016) for peptide ligation. It is noteworthy that ascorbate is still incapable of preventing the homolytic cleavage of selenol at more activated positions (i.e. deselenization at β-seleno Asp and γ-seleno Glu residues) (Mitchell et al., 2017). Also harnessing the complementary reactivity between phosphine and selenol, Malins (Malins et al., 2015b) and Dery et al. (Dery et al., 2015) have independently developed different oxidative deselenization protocols that effectively transform Sec to Ser at the ligation junction after ligation, which allow for retrosynthetic disconnection at X-Ser junctions. Recently, Metanis and coworkers have attempted to utilize the unique reactivity of γ-selenoLys to establish an isopeptide ligation−deselenization method for chemical synthesis of proteins modified by SUMO or ubiquitin (Dardashti et al., 2020). However, the resulting isopeptide ligation proceeded very slowly despite adding excess amounts of TCEP and sodium ascorbate. The authors proposed that the higher pKa of the ε-amine in Lys prevents transesterification to occur in neutral ligation buffer; however, the underlying mechanism remains to be investigated.

Payne and coworkers reported an additive-free ligation method (dubbed DSL) (Mitchell et al., 2015) that could potentially overcome the bottlenecks in selenium-mediated peptide ligation: first, the approach enables the reaction between a peptide diselenide and a peptide bearing C-terminal phenyl selenoester to complete within minutes without any reductive additives, which compares favorably to the rate of a respective NCL reaction. Second, this chemistry exhibits high tolerance to acidic pH and enables the ligation reaction to proceed at pH 3–7, which could effectively suppress hydrolysis of selenoesters. Third, the selenol auxiliary can be selectively removed *in situ* (using excess amounts of TCEP and DTT) after DSL without the need for intermediary purification. With these salient features and the demonstrated orthogonality between selenol and other proteogenic functionalities, it is anticipated that this methodology will find wide application in the research community of chemical protein synthesis. Recently, the harmony between NCL and DSL has been proven in the total synthesis of phosphorylated insulin-like growth factor binding protein 2 (Premdjee et al., 2021).

In the original forms, both NCL and DSL reactions require millimolar concentrations of ligation fragments to achieve productive kinetics and to afford high-yielding synthesis, however proceeding sluggishly at micromolar concentrations. Harnessing the superior reactivities of phenyl selenoester and Sec in reducing environment, Payne and co-workers reported a reductive diselenide-selenoester ligation (rDSL) method that enables efficient peptide ligation down to 50 nM (Chisholm et al., 2020). In addition, the authors demonstrated that an efficient photodeselenization process can be performed after ligation to rapidly afford the target polypeptides in a one-pot manner. The power of the rDSL-photodeselenization manifold has been showcased in the chemical synthesis of a lipidated peptide therapeutic (tesamorelin) and two palmitylated variants of an integral membrane protein (FXYD1) without the aid of solubility tags and hybridizing templates. With the global endeavor to develop more efficient strategies to access selenylated amino acids, it is expected that the scope of the DSL and rDSL methodologies will be continuously expanded and may allow one to exploit the scope beyond total synthesis of proteins (Sayers et al., 2018b).

## Conclusion

Recent advances of peptide ligation chemistry have greatly enhanced our capacity to produce native proteins and even peptide therapeutics with defined, bespoke modifications. In particular, these new methods have expanded the scope of NCL beyond the need of adequately positioned Cys residues within a protein target, including the successes in preparing the thiol derived variants of Phe, Val, Lys, Thr, Ile, Leu, Pro, Arg, Asn, Gln, Asp, Glu, and Trp (Table 1), and the selenyl variants of Phe, Lys, Pro, Asp, and Glu (Table 2), and the recent approach to access a large variety of β-thiolated/selenylated amino acids based on photoredox-catalyzed asymmetric Giese reactions (Table 1 and 2). Integrating these important artificial amino acids into advanced ligation–desulfurization or ligation−deselenization methodologies has provided a viable alternative to access homogenously modified proteins in highly efficient manner and has advanced our knowledge on the roles of PTMs in protein function, activity, and structure. While such synthetic approaches cannot compete with the ultra-large protein libraries generated through phage display (Salmond and Fineran, 2015; Alfaleh et al., 2020), mRNA display (Murakami et al., 2006; Goto et al., 2011) and codon expansion and reprogramming technologies (Chin, 2014; Wals and Ovaa, 2014), the latest ligation techniques offer an exciting opportunity to establish novel protein medicinal chemistry programs through constructing focused libraries, wherein distinct PTMs and bespoke modifications can be incorporated site-specifically allowing one to explore a unique and important chemical space in protein therapeutics. Furthermore, the fast kinetics and chemical specificity of the recently established selenium-based ligation technologies provide a potential avenue to engineer proteins at low concentrations in non-denaturing buffer (i.e., physiological buffer), which may offer an exciting possibility for other chemical biology applications, including cell-surface protein engineering, rapid antigen testing and antibody-drug conjugation.

**TABLE 1:** Thiol derived amino acids and the relevant synthesis examples described in this article.

Thiolated amino acid	Synthetic strategy	Peptide and protein synthesis example	References(s)
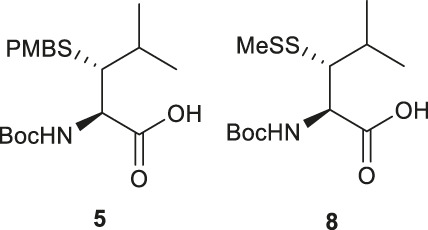	1. Aziridine ring opening	ATAD2 bromodomain region	Harpaz et al. (2010) Tan et al. (2010) Creech et al. (2014)
	2. Nucleophilic displacement		
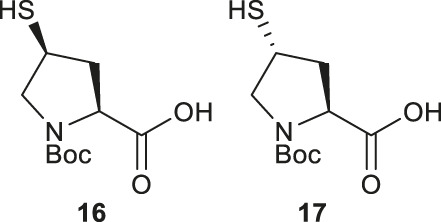	Nucleophilic displacement	1. Rat neuromedin U	Ding et al. (2011) Townsend et al. (2012)
		2. hEPO (79–166) Glycopeptide	
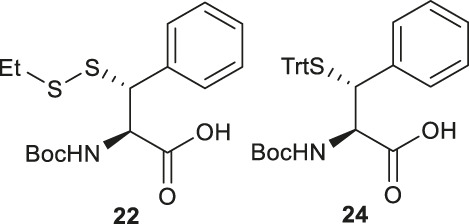	1. Nucleophilic displacement	1. Fragment of Augurin	Malins et al. (2015a) Wang et al. (2020a)
	2. Garner’s aldehyde	2. Glycosylated human IFN-γ	
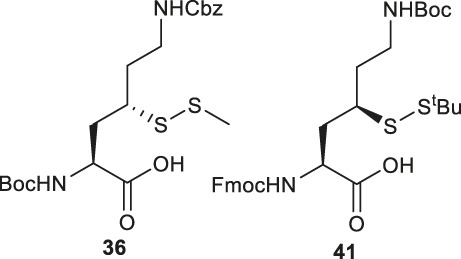	Nucleophilic displacement	1. Ubiquitinated peptide	Pasunooti et al. (2009) Merkx et al. (2013)
		2. Diubiquitin and tetraubiquitin	
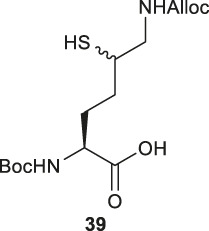	Nucleophilic conjugate addition	1. Diubiquitin	Ajish Kumar et al. (2009) Siman et al. (2013)
		2. Site-specific ubiquitinated H2B	
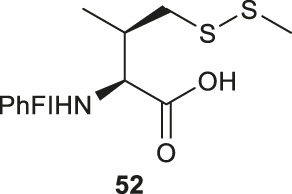	Nucleophilic displacement	Model peptides	Chen et al. (2008)
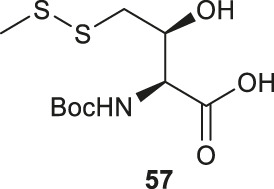	Nucleophilic displacement	Model peptides	Chen et al. (2010)
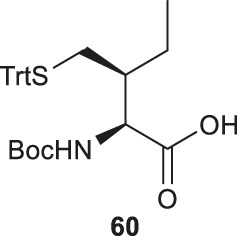	C (sp^3^)-H activation	*Xenopus* H3	Pasunooti et al. (2016)
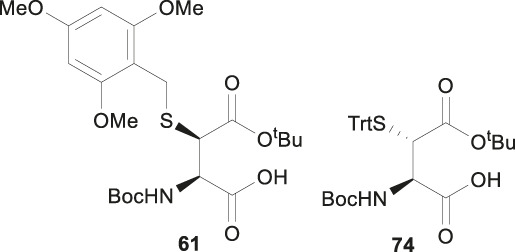	1. Electrophilic sulfenylation	1. Extracellular N-terminal domain of CXCR4	Watson et al. (2018) Thompson et al. (2013) Guan et al. (2013) Li et al. (2021b)
	2. Nucleophilic displacement	2. Sulfated anopheline proteins	
		3. Glycosylated IL-17A	
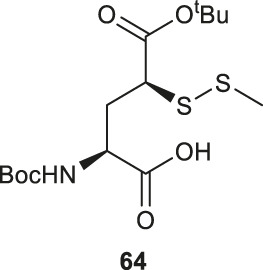	Electrophilic sulfenylation	Teriparatide	Cergol et al. (2014)
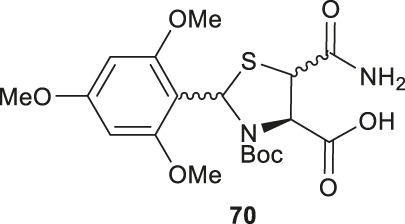	Electrophilic sulfenylation	Enfuvirtide	Sayers et al. (2015)
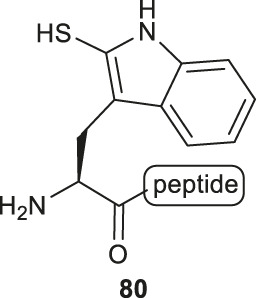	Electrophilic sulfenylation	Extracellular N-terminal domain of CXCR1	Malins et al. (2014)
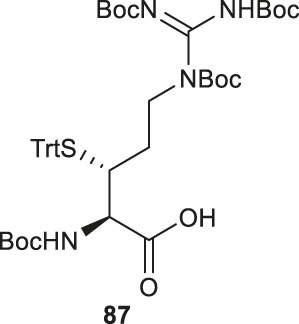	Garner’s aldehyde	Glycosylated extracellular domain of MUC1	Malins et al. (2013)
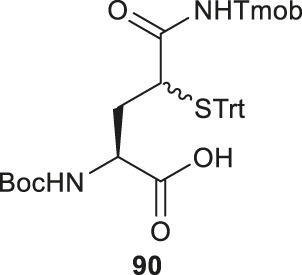	Passerini three-component reaction	Model peptides	Siman et al. (2012)
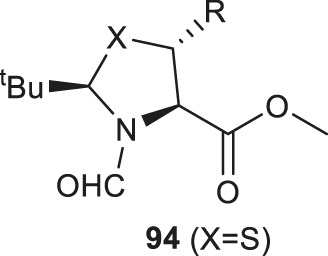	Photoredox-catalyzed asymmetric	Model peptides	Yin et al. (2020)
	Giese reaction		
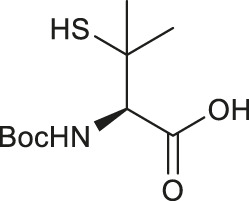	Commercially available	Tri-phosphorylated C-terminal Tau	Reimann et al. (2015) Haase et al. (2008)

**TABLE 2:** Selenol derived amino acids and the relevant synthesis examples described in this article.

Selenolated amino acid	Synthetic strategy	Peptide and protein synthesis example	References(s)
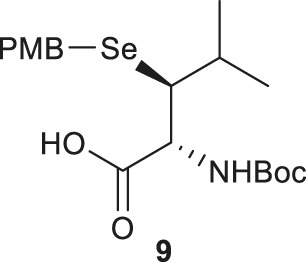	Garner’s aldehyde	Sulfated UL22A	Wang et al. (2017)
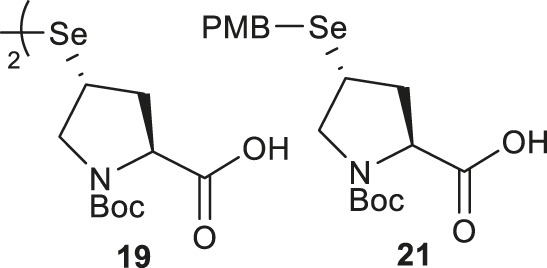	Nucleophilic displacement	1. Hydroxy-proline model peptide	Townsend et al. (2012) Sayers et al. (2018a)
		2. SMR3B	
		3. Lumbricin-1	
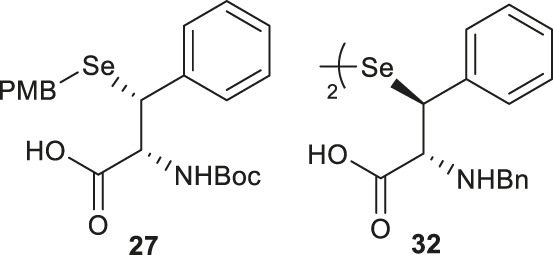	1. Nucleophilic displacement	Model peptides	Malins and Payne, (2012)
	2. Garner’s aldehyde		
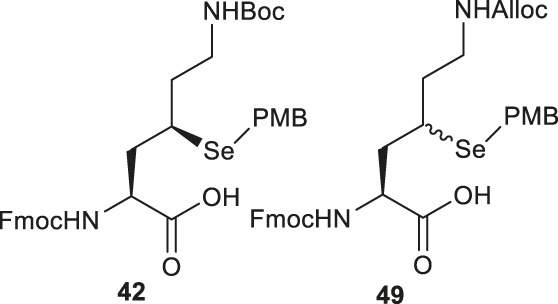	1. Nucleophilic displacement	SUMOylated GCK	Dardashti et al. (2020)
	2. Nucleophilic conjugate addition		
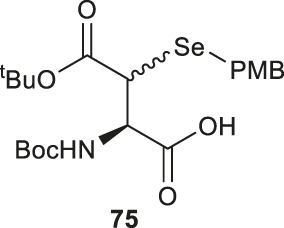	Electrophilic sulfenylation	1.Sulfated Hya1-3	Watson et al. (2019) Mitchell et al. (2017)
		2. Selenoprotein K	
		3. And82, And310 and MDL2	
		4. non-modified Hya2-4	
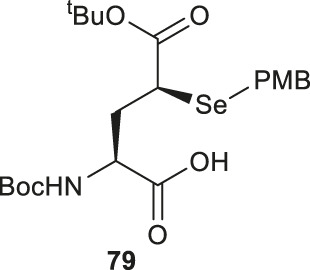	Electrophilic sulfenylation	Model peptides	Mitchell et al. (2017)
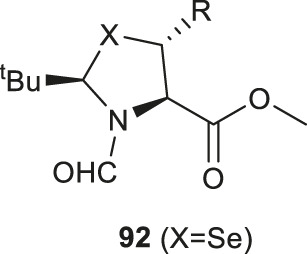	Photoredox-catalyzed asymmetric Giese reaction	Cytpchrome *c* Oxidase subunit protein 7C	Yin et al. (2020)
		Oxytocin analogues	

## References

[B1] Agten S. M. Watson E. E. Ripoll‐Rozada J. Dowman L. J. Wu M. C. L. Alwis I. (2021). Potent Trivalent Inhibitors of Thrombin through Hybridization of Salivary Sulfopeptides from Hematophagous Arthropods. Angew. Chem. Int. Ed. 60 (10), 5348–5356. 10.1002/anie.202015127 33345438

[B2] Ajish Kumar K. S. Haj-Yahya M. Olschewski D. Lashuel H. A. Brik A. (2009). Highly Efficient and Chemoselective Peptide Ubiquitylation. Angew. Chem. Int. Ed. 48 (43), 8090–8094. 10.1002/anie.200902936 19780082

[B3] Alfaleh M. A. Alsaab H. O. Mahmoud A. B. Alkayyal A. A. Jones M. L. Mahler S. M. (2020). Phage Display Derived Monoclonal Antibodies: From Bench to Bedside. Front. Immunol. 11, 1986. 10.3389/fimmu.2020.01986 32983137 PMC7485114

[B4] Arnold F. H. (2018). Directed Evolution: Bringing New Chemistry to Life. Angew. Chem. Int. Ed. 57 (16), 4143–4148. 10.1002/anie.201708408 PMC5901037 29064156

[B5] Bang D. Pentelute B. L. Kent S. B. H. (2006). Kinetically Controlled Ligation for the Convergent Chemical Synthesis of Proteins. Angew. Chem. Int. Ed. 45 (24), 3985–3988. 10.1002/anie.200600702 16639756

[B6] Barnes N. G. Nyandoro K. Jin H. Macmillan D. (2021). Rapid Access to Asp/Glu Sidechain Hydrazides as Thioester Precursors for Peptide Cyclization and Glycosylation. Chem. Commun. 57 (8), 1006–1009. 10.1039/d0cc07404g 33399597

[B7] Buettner C. S. Willcox D. Chappell B. G. N. Gaunt M. J. (2019). Mechanistic Investigation into the C(sp3)-H Acetoxylation of Morpholinones. Chem. Sci. 10 (1), 83–89. 10.1039/c8sc03434f 30713620 PMC6331033

[B8] Cergol K. M. Thompson R. E. Malins L. R. Turner P. Payne R. J. (2014). One-Pot Peptide Ligation-Desulfurization at Glutamate. Org. Lett. 16 (1), 290–293. 10.1021/ol403288n 24294973

[B9] Chen J. Wan Q. Yuan Y. Zhu J. Danishefsky S. J. (2008). Native Chemical Ligation at Valine: A Contribution to Peptide and Glycopeptide Synthesis. Angew. Chem. Int. Ed. 47 (44), 8521–8524. 10.1002/anie.200803523 PMC2756581 18833563

[B10] Chen J. Wang P. Zhu J. Wan Q. Danishefsky S. J. (2010). A Program for Ligation at Threonine Sites: Application to the Controlled Total Synthesis of Glycopeptides. Tetrahedron 66 (13), 2277–2283. 10.1016/j.tet.2010.01.067 20798898 PMC2925322

[B11] Chin J. W. (2014). Expanding and Reprogramming the Genetic Code of Cells and Animals. Annu. Rev. Biochem. 83 (1), 379–408. 10.1146/annurev-biochem-060713-035737 24555827

[B12] Chisholm T. S. Kulkarni S. S. Hossain K. R. Cornelius F. Clarke R. J. Payne R. J. (2020). Peptide Ligation at High Dilution via Reductive Diselenide-Selenoester Ligation. J. Am. Chem. Soc. 142 (2), 1090–1100. 10.1021/jacs.9b12558 31840988

[B13] Conibear A. C. Watson E. E. Payne R. J. Becker C. F. W. (2018). Native Chemical Ligation in Protein Synthesis and Semi-synthesis. Chem. Soc. Rev. 47 (24), 9046–9068. 10.1039/c8cs00573g 30418441

[B14] Creech G. S. Paresi C. Li Y.-M. Danishefsky S. J. (2014). Chemical Synthesis of the ATAD2 Bromodomain. Proc. Natl. Acad. Sci. 111 (8), 2891–2896. 10.1073/pnas.1400556111 24516155 PMC3939912

[B15] Crich D. Banerjee A. (2007). Native Chemical Ligation at Phenylalanine. J. Am. Chem. Soc. 129 (33), 10064–10065. 10.1021/ja072804l 17658806

[B16] Dardashti R. N. Kumar S. Sternisha S. M. Reddy P. S. Miller B. G. Metanis N. (2020). Selenolysine: A New Tool for Traceless Isopeptide Bond Formation. Chem. Eur. J. 26 (22), 4952–4957. 10.1002/chem.202000310 31960982 PMC7184786

[B17] Dawson P. E. Muir T. W. Clark-Lewis I. Kent S. B. H. (1994). Synthesis of Proteins by Native Chemical Ligation. Science 266 (5186), 776–779. 10.1126/science.7973629 7973629

[B18] Dery S. Reddy P. S. Dery L. Mousa R. Dardashti R. N. Metanis N. (2015). Insights into the Deselenization of Selenocysteine into Alanine and Serine. Chem. Sci. 6 (11), 6207–6212. 10.1039/c5sc02528a 30090236 PMC6054048

[B19] Ding H. Shigenaga A. Sato K. Morishita K. Otaka A. (2011). Dual Kinetically Controlled Native Chemical Ligation Using a Combination of Sulfanylproline and Sulfanylethylanilide Peptide. Org. Lett. 13 (20), 5588–5591. 10.1021/ol202316v 21916452

[B20] Durek T. Alewood P. F. (2011). Preformed Selenoesters Enable Rapid Native Chemical Ligation at Intractable Sites. Angew. Chem. Int. Ed. 50 (50), 12042–12045. 10.1002/anie.201105512 21997950

[B21] Easton C. J. Hutton C. A. Roselt P. D. Tiekink E. R. T. (1994). Stereocontrolled Synthesis of β-hydroxyphenylalanine and β-hydroxytyrosine Derivatives. Tetrahedron 50 (24), 7327–7340. 10.1016/s0040-4020(01)85256-x

[B22] Fang G.-M. Li Y.-M. Shen F. Huang Y.-C. Li J.-B. Lin Y. (2011). Protein Chemical Synthesis by Ligation of Peptide Hydrazides. Angew. Chem. Int. Ed. 50 (33), 7645–7649. 10.1002/anie.201100996 21648030

[B23] Flavell R. R. Muir T. W. (2009). Expressed Protein Ligation (EPL) in the Study of Signal Transduction, Ion Conduction, and Chromatin Biology. Acc. Chem. Res. 42 (1), 107–116. 10.1021/ar800129c 18939858

[B24] Flood D. T. Hintzen J. C. J. Bird M. J. Cistrone P. A. Chen J. S. Dawson P. E. (2018). Leveraging the Knorr Pyrazole Synthesis for the Facile Generation of Thioester Surrogates for Use in Native Chemical Ligation. Angew. Chem. Int. Ed. 57 (36), 11634–11639. 10.1002/anie.201805191 PMC6126375 29908104

[B25] Garner P. (1984). Stereocontrolled Addition to a Penaldic Acid Equivalent: an Asymmetric of -β-Hydroxy-L-Glutamic Acid. Tetrahedron Lett. 25 (51), 5855–5858. 10.1016/s0040-4039(01)81703-2

[B26] Gieselman M. D. Xie L. Van Der Donk W. A. (2001). Synthesis of a Selenocysteine-Containing Peptide by Native Chemical Ligation. Org. Lett. 3 (9), 1331–1334. 10.1021/ol015712o 11348227

[B27] Goto Y. Katoh T. Suga H. (2011). Flexizymes for Genetic Code Reprogramming. Nat. Protoc. 6 (6), 779–790. 10.1038/nprot.2011.331 21637198

[B28] Guan X. Drake M. R. Tan Z. (2013). Total Synthesis of Human Galanin-like Peptide through an Aspartic Acid Ligation. Org. Lett. 15 (24), 6128–6131. 10.1021/ol402984r 24266801

[B29] Haase C. Rohde H. Seitz O. (2008). Native Chemical Ligation at Valine. Angew. Chem. Int. Ed. 47 (36), 6807–6810. 10.1002/anie.200801590 18626881

[B30] Hackenberger C. P. R. (2006). The Reduction of Oxidized Methionine Residues in Peptide Thioesters with NH4I-Me2S. Org. Biomol. Chem. 4 (11), 2291–2295. 10.1039/b603543d 16729139

[B31] Hackeng T. M. Griffin J. H. Dawson P. E. (1999). Protein Synthesis by Native Chemical Ligation: Expanded Scope by Using Straightforward Methodology. Proc. Natl. Acad. Sci. 96 (18), 10068–10073. 10.1073/pnas.96.18.10068 10468563 PMC17843

[B32] Harpaz Z. Siman P. Kumar K. S. A. Brik A. (2010). Protein Synthesis Assisted by Native Chemical Ligation at Leucine. Chem. Eur. J. Chem. Bio. 11 (9), 1232–1235. 10.1002/cbic.201000168 20437446

[B33] Hofmann R. M. Muir T. W. (2002). Recent Advances in the Application of Expressed Protein Ligation to Protein Engineering. Curr. Opin. Biotechnol. 13 (4), 297–303. 10.1016/s0958-1669(02)00326-9 12323349

[B34] Hondal R. J. Nilsson B. L. Raines R. T. (2001). Selenocysteine in Native Chemical Ligation and Expressed Protein Ligation. J. Am. Chem. Soc. 123 (21), 5140–5141. 10.1021/ja005885t 11457362

[B35] Hsieh Y. S. Y. Wijeyewickrema L. C. Wilkinson B. L. Pike R. N. Payne R. J. (2014). Total Synthesis of Homogeneous Variants of Hirudin P6: A Post-Translationally Modified Anti-thrombotic Leech-Derived Protein. Angew. Chem. Int. Ed. 53 (15), 3947–3951. 10.1002/anie.201310777 24615823

[B36] Jbara M. Guttmann-Raviv N. Maity S. K. Ayoub N. Brik A. (2017). Total Chemical Synthesis of Methylated Analogues of Histone 3 Revealed KDM4D as a Potential Regulator of H3K79me3. Bioorg. Med. Chem. 25 (18), 4966–4970. 10.1016/j.bmc.2017.04.015 28434780

[B37] Jbara M. Maity S. K. Morgan M. Wolberger C. Brik A. (2016). Chemical Synthesis of Phosphorylated Histone H2A at Tyr57 Reveals Insight into the Inhibition Mode of the SAGA Deubiquitinating Module. Angew. Chem. Int. Ed. 55 (16), 4972–4976. 10.1002/anie.201600638 PMC4944388 26960207

[B38] Kilic S. Boichenko I. Lechner C. C. Fierz B. (2018). A Bi-terminal Protein Ligation Strategy to Probe Chromatin Structure during DNA Damage. Chem. Sci. 9 (15), 3704–3709. 10.1039/c8sc00681d 29780501 PMC5935033

[B39] Kumar K. S. A. Bavikar S. N. Spasser L. Moyal T. Ohayon S. Brik A. (2011). Total Chemical Synthesis of a 304 Amino Acid K48-Linked Tetraubiquitin Protein. Angew. Chem. Int. Ed. 50 (27), 6137–6141. 10.1002/anie.201101920 21591043

[B40] Kumar K. S. A. Spasser L. Erlich L. A. Bavikar S. N. Brik A. (2010). Total Chemical Synthesis of Di-ubiquitin Chains. Angew. Chem. Int. Edition 49 (48), 9126–9131. 10.1002/anie.201003763 20815002

[B41] Li H. Zhang J. An C. Dong S. (2021b). Probing N-Glycan Functions in Human Interleukin-17A Based on Chemically Synthesized Homogeneous Glycoforms. J. Am. Chem. Soc. 143 (7), 2846–2856. 10.1021/jacs.0c12448 33577335

[B42] Li J.-B. Qi Y.-K. He Q.-Q. Ai H.-S. Liu S.-l. Wang J.-X. (2018). Chemically Synthesized Histone H2A Lys13 Di-ubiquitination Promotes Binding of 53BP1 to Nucleosomes. Cell Res 28 (2), 257–260. 10.1038/cr.2017.157 29243734 PMC5799816

[B43] Li Y. Heng J. Sun D. Zhang B. Zhang X. Zheng Y. (2021a). Chemical Synthesis of a Full-Length G-Protein-Coupled Receptor β2-Adrenergic Receptor with Defined Modification Patterns at the C-Terminus. J. Am. Chem. Soc. 143 (42), 17566–17576. 10.1021/jacs.1c07369 34663067

[B44] Malins L. R. Cergol K. M. Payne R. J. (2014). Chemoselective Sulfenylation and Peptide Ligation at Tryptophan. Chem. Sci. 5 (1), 260–266. 10.1039/c3sc51497h

[B45] Malins L. R. Cergol K. M. Payne R. J. (2013). Peptide Ligation-Desulfurization Chemistry at Arginine. ChemBioChem 14 (5), 559–563. 10.1002/cbic.201300049 23426906

[B46] Malins L. R. Giltrap A. M. Dowman L. J. Payne R. J. (2015a). Synthesis of β-Thiol Phenylalanine for Applications in One-Pot Ligation-Desulfurization Chemistry. Org. Lett. 17 (9), 2070–2073. 10.1021/acs.orglett.5b00597 25860301

[B47] Malins L. R. Mitchell N. J. McGowan S. Payne R. J. (2015b). Oxidative Deselenization of Selenocysteine: Applications for Programmed Ligation at Serine. Angew. Chem. Int. Ed. 54 (43), 12716–12721. 10.1002/anie.201504639 26384718

[B48] Malins L. R. Payne R. J. (2012). Synthesis and Utility of β-Selenol-Phenylalanine for Native Chemical Ligation-Deselenization Chemistry. Org. Lett. 14 (12), 3142–3145. 10.1021/ol3012265 22642500

[B49] Marin J. Didierjean C. Aubry A. Casimir J.-R. Briand J.-P. Guichard G. (2004). Synthesis of Enantiopure 4-Hydroxypipecolate and 4-Hydroxylysine Derivatives from a Common 4,6-Dioxopiperidinecarboxylate Precursor. J. Org. Chem. 69 (1), 130–141. 10.1021/jo0353886 14703388

[B50] Merkx R. De Bruin G. Kruithof A. Van Den Bergh T. Snip E. Lutz M. (2013). Scalable Synthesis of γ-thiolysine Starting from Lysine and a Side by Side Comparison with δ-thiolysine in Non-enzymatic Ubiquitination. Chem. Sci. 4 (12), 4494. 10.1039/c3sc51599k

[B51] Metanis N. Keinan E. Dawson P. E. (2010). Traceless Ligation of Cysteine Peptides Using Selective Deselenization. Angew. Chem. Int. Edition 49 (39), 7049–7053. 10.1002/anie.201001900 PMC4459706 20715234

[B52] Mitchell N. J. Malins L. R. Liu X. Thompson R. E. Chan B. Radom L. (2015). Rapid Additive-free Selenocystine-Selenoester Peptide Ligation. J. Am. Chem. Soc. 137 (44), 14011–14014. 10.1021/jacs.5b07237 26487084

[B53] Mitchell N. J. Sayers J. Kulkarni S. S. Clayton D. Goldys A. M. Ripoll-Rozada J. (2017). Accelerated Protein Synthesis via One-Pot Ligation-Deselenization Chemistry. Chem 2 (5), 703–715. 10.1016/j.chempr.2017.04.003

[B54] Moura A. Savageau M. A. Alves R. (2013). Relative Amino Acid Composition Signatures of Organisms and Environments. PLoS ONE 8 (10), e77319. 10.1371/journal.pone.0077319 24204807 PMC3808408

[B55] Muir T. W. (2003). Semisynthesis of Proteins by Expressed Protein Ligation. Annu. Rev. Biochem. 72 (1), 249–289. 10.1146/annurev.biochem.72.121801.161900 12626339

[B56] Muir T. W. Sondhi D. Cole P. A. (1998). Expressed Protein Ligation: A General Method for Protein Engineering. Proc. Natl. Acad. Sci. 95 (12), 6705–6710. 10.1073/pnas.95.12.6705 9618476 PMC22605

[B57] Murakami H. Ohta A. Ashigai H. Suga H. (2006). A Highly Flexible tRNA Acylation Method for Non-natural Polypeptide Synthesis. Nat. Methods 3 (5), 357–359. 10.1038/nmeth877 16628205

[B58] Murakami M. Kiuchi T. Nishihara M. Tezuka K. Okamoto R. Izumi M. (2016). Chemical Synthesis of Erythropoietin Glycoforms for Insights into the Relationship between Glycosylation Pattern and Bioactivity. Sci. Adv. 2 (1), e1500678. 10.1126/sciadv.1500678 26824070 PMC4730857

[B59] Nishimoto Y. Okita A. Yasuda M. Baba A. (2012). Synthesis of a Wide Range of Thioethers by Indium Triiodide Catalyzed Direct Coupling between Alkyl Acetates and Thiosilanes. Org. Lett. 14 (7), 1846–1849. 10.1021/ol300450j 22428542

[B60] Nuijens T. Toplak A. Schmidt M. Ricci A. Cabri W. (2019). Natural Occurring and Engineered Enzymes for Peptide Ligation and Cyclization. Front. Chem. 7 (829), 829. 10.3389/fchem.2019.00829 31850317 PMC6895249

[B61] Offer J. (2010). Native Chemical Ligation with Nα Acyl Transfer Auxiliaries. Biopolymers 94 (4), 530–541. 10.1002/bip.21455 20593473

[B62] Pan M. Zheng Q. Gao S. Qu Q. Yu Y. Wu M. (2019). Chemical Synthesis of Structurally Defined Phosphorylated Ubiquitins Suggests Impaired Parkin Activation by Phosphorylated Ubiquitins with a Non-phosphorylated Distal Unit. CCS Chem. 1 (5), 476–489. 10.31635/ccschem.019.20190001

[B63] Passiniemi M. Koskinen A. M. (2013). Garner's Aldehyde as a Versatile Intermediate in the Synthesis of Enantiopure Natural Products. Beilstein J. Org. Chem. 9, 2641–2659. 10.3762/bjoc.9.300 24367429 PMC3869249

[B64] Pasunooti K. K. Banerjee B. Yap T. Jiang Y. Liu C.-F. (2015). Auxiliary-Directed Pd-Catalyzed γ-C(sp3)-H Bond Activation of α-Aminobutanoic Acid Derivatives. Org. Lett. 17 (24), 6094–6097. 10.1021/acs.orglett.5b03118 26634407

[B65] Pasunooti K. K. Yang R. Banerjee B. Yap T. Liu C.-F. (2016). 5-Methylisoxazole-3-carboxamide-Directed Palladium-Catalyzed γ-C(sp3)-H Acetoxylation and Application to the Synthesis of γ-Mercapto Amino Acids for Native Chemical Ligation. Org. Lett. 18 (11), 2696–2699. 10.1021/acs.orglett.6b01160 27218276

[B66] Pasunooti K. K. Yang R. Vedachalam S. Gorityala B. K. Liu C.-F. Liu X.-W. (2009). Synthesis of 4-Mercapto-L-Lysine Derivatives: Potential Building Blocks for Sequential Native Chemical Ligation. Bioorg. Med. Chem. Lett. 19 (22), 6268–6271. 10.1016/j.bmcl.2009.09.107 19833511

[B67] Pollock S. B. Kent S. B. H. (2011). An Investigation into the Origin of the Dramatically Reduced Reactivity of Peptide-Prolyl-Thioesters in Native Chemical Ligation. Chem. Commun. 47 (8), 2342–2344. 10.1039/c0cc04120c 21173985

[B68] Premdjee B. Andersen A. S. Larance M. Conde-Frieboes K. W. Payne R. J. (2021). Chemical Synthesis of Phosphorylated Insulin-like Growth Factor Binding Protein 2. J. Am. Chem. Soc. 143 (14), 5336–5342. 10.1021/jacs.1c02280 33797881

[B69] Qi Y.-K. Si Y.-Y. Du S.-S. Liang J. Wang K.-W. Zheng J.-S. (2019). Recent Advances in the Chemical Synthesis and Semi-synthesis of Poly-Ubiquitin-Based Proteins and Probes. Sci. China Chem. 62 (3), 299–312. 10.1007/s11426-018-9401-8

[B70] Qu W. Zha Z. Ploessl K. Lieberman B. P. Zhu L. Wise D. R. (2011). Synthesis of Optically Pure 4-Fluoro-Glutamines as Potential Metabolic Imaging Agents for Tumors. J. Am. Chem. Soc. 133 (4), 1122–1133. 10.1021/ja109203d 21190335

[B71] Quaderer R. Sewing A. Hilvert D. (2001). Selenocysteine‐Mediated Native Chemical Ligation. Helvetica Chim. Acta 84 (5), 1197–1206. 10.1002/1522-2675(20010516)84:5<1197:aid-hlca1197>3.0.co;2-#

[B72] Rashid Baig N. B. Chandrakala R. N. Sudhir V. S. Chandrasekaran S. (2010). Synthesis of Unnatural Selenocystines and β-Aminodiselenides via Regioselective Ring-Opening of Sulfamidates Using a Sequential, One-Pot, Multistep Strategy. J. Org. Chem. 75 (9), 2910–2921. 10.1021/jo1001388 20392054

[B73] Reddy P. S. Dery S. Metanis N. (2016). Chemical Synthesis of Proteins with Non-strategically Placed Cysteines Using Selenazolidine and Selective Deselenization. Angew. Chem. 128 (3), 1004–1007. 10.1002/ange.201509378 26636774

[B74] Reimann O. Glanz M. Hackenberger C. P. R. (2015). Native Chemical Ligation between Asparagine and Valine: Application and Limitations for the Synthesis of Tri-phosphorylated C-Terminal Tau. Bioorg. Med. Chem. 23 (12), 2890–2894. 10.1016/j.bmc.2015.03.028 25882528

[B75] Salmond G. P. C. Fineran P. C. (2015). A century of the Phage: Past, Present and Future. Nat. Rev. Microbiol. 13 (12), 777–786. 10.1038/nrmicro3564 26548913

[B76] Sayers J. Karpati P. M. T. Mitchell N. J. Goldys A. M. Kwong S. M. Firth N. (2018a). Construction of Challenging Proline-Proline Junctions via Diselenide-Selenoester Ligation Chemistry. J. Am. Chem. Soc. 140 (41), 13327–13334. 10.1021/jacs.8b07877 30239198

[B77] Sayers J. Payne R. J. Winssinger N. (2018b). Peptide Nucleic Acid-Templated Selenocystine-Selenoester Ligation Enables Rapid miRNA Detection. Chem. Sci. 9 (4), 896–903. 10.1039/c7sc02736b 29629156 PMC5873163

[B78] Sayers J. Thompson R. E. Perry K. J. Malins L. R. Payne R. J. (2015). Thiazolidine-Protected β-Thiol Asparagine: Applications in One-Pot Ligation-Desulfurization Chemistry. Org. Lett. 17 (19), 4902–4905. 10.1021/acs.orglett.5b02468 26398220

[B79] Schmidt M. Toplak A. Quaedflieg P. J. Nuijens T. (2017). Enzyme-mediated Ligation Technologies for Peptides and Proteins. Curr. Opin. Chem. Biol. 38, 1–7. 10.1016/j.cbpa.2017.01.017 28229906

[B80] Seenaiah M. Jbara M. Mali S. M. Brik A. (2015). Convergent versus Sequential Protein Synthesis: The Case of Ubiquitinated and Glycosylated H2B. Angew. Chem. Int. Ed. 54 (42), 12374–12378. 10.1002/anie.201503309 26079184

[B81] Shabani S. Wu Y. Ryan H. G. Hutton C. A. (2021). Progress and Perspectives on Directing Group-Assisted Palladium-Catalysed C-H Functionalisation of Amino Acids and Peptides. Chem. Soc. Rev. 50 (16), 9278–9343. 10.1039/d0cs01441a 34254063

[B82] Shibata N. Baldwin J. E. Jacobs A. Wood M. E. (1996). Electrophilic Sulfenylation in a Stereocontrolled Synthesis of Protected (2R,3R)-3-Mercaptoaspartic Acid from -aspartic Acid. Tetrahedron 52 (39), 12839–12852. 10.1016/0040-4020(96)00765-x

[B83] Shogren-Knaak M. A. Fry C. J. Peterson C. L. (2003). A Native Peptide Ligation Strategy for Deciphering Nucleosomal Histone Modifications. J. Biol. Chem. 278 (18), 15744–15748. 10.1074/jbc.m301445200 12595522

[B84] Siman P. Karthikeyan S. V. Brik A. (2012). Native Chemical Ligation at Glutamine. Org. Lett. 14 (6), 1520–1523. 10.1021/ol300254y 22360701

[B85] Siman P. Karthikeyan S. V. Nikolov M. Fischle W. Brik A. (2013). Convergent Chemical Synthesis of Histone H2B Protein for the Site-specific Ubiquitination at Lys34. Angew. Chem. Int. Ed. 52 (31), 8059–8063. 10.1002/anie.201303844 23794525

[B86] Tan Z. Shang S. Danishefsky S. J. (2010). Insights into the Finer Issues of Native Chemical Ligation: An Approach to Cascade Ligations. Angew. Chem. Int. Ed. 49 (49), 9500–9503. 10.1002/anie.201005513 PMC3199326 21053233

[B87] Thompson R. E. Chan B. Radom L. Jolliffe K. A. Payne R. J. (2013). Chemoselective Peptide Ligation-Desulfurization at Aspartate. Angew. Chem. Int. Ed. 52 (37), 9723–9727. 10.1002/anie.201304793 23893778

[B88] Thompson R. E. Liu X. Alonso-García N. Pereira P. J. B. Jolliffe K. A. Payne R. J. (2014). Trifluoroethanethiol: An Additive for Efficient One-Pot Peptide Ligation−Desulfurization Chemistry. J. Am. Chem. Soc. 136 (23), 8161–8164. 10.1021/ja502806r 24873761

[B89] Thompson R. E. Liu X. Ripoll-Rozada J. Alonso-García N. Parker B. L. Pereira P. J. B. (2017). Tyrosine Sulfation Modulates Activity of Tick-Derived Thrombin Inhibitors. Nat. Chem 9 (9), 909–917. 10.1038/nchem.2744 28837178

[B90] Thompson R. E. Muir T. W. (2020). Chemoenzymatic Semisynthesis of Proteins. Chem. Rev. 120 (6), 3051–3126. 10.1021/acs.chemrev.9b00450 31774265 PMC7101271

[B91] Townsend S. D. Tan Z. Dong S. Shang S. Brailsford J. A. Danishefsky S. J. (2012). Advances in Proline Ligation. J. Am. Chem. Soc. 134 (8), 3912–3916. 10.1021/ja212182q 22332757 PMC3306728

[B92] Van Der Heden Van Noort G. J. Kooij R. Elliott P. R. Komander D. Ovaa H. (2017). Synthesis of Poly-Ubiquitin Chains Using a Bifunctional Ubiquitin Monomer. Org. Lett. 19 (24), 6490–6493. 10.1021/acs.orglett.7b03085 29172548 PMC5735377

[B93] Wals K. Ovaa H. (2014). Unnatural Amino Acid Incorporation in *E. coli*: Current and Future Applications in the Design of Therapeutic Proteins. Front. Chem. 2 (15), 15. 10.3389/fchem.2014.00015 24790983 PMC3982533

[B94] Wan Q. Danishefsky S. J. (2007). Free-Radical-Based, Specific Desulfurization of Cysteine: A Powerful Advance in the Synthesis of Polypeptides and Glycopolypeptides. Angew. Chem. Int. Ed. 46 (48), 9248–9252. 10.1002/anie.200704195 18046687

[B95] Wang P. Dong S. Shieh J.-H. Peguero E. Hendrickson R. Moore M. A. S. (2013). Erythropoietin Derived by Chemical Synthesis. Science 342 (6164), 1357–1360. 10.1126/science.1245095 24337294 PMC4080428

[B96] Wang X. Ashhurst A. S. Dowman L. J. Watson E. E. Li H. Y. Fairbanks A. J. (2020). Total Synthesis of Glycosylated Human Interferon-γ. Org. Lett. 22 (17), 6863–6867. 10.1021/acs.orglett.0c02401 32830985

[B97] Wang X. Corcilius L. Premdjee B. Payne R. J. (2020). Synthesis and Utility of β-Selenophenylalanine and β-Selenoleucine in Diselenide-Selenoester Ligation. J. Org. Chem. 85 (3), 1567–1578. 10.1021/acs.joc.9b02665 31840993

[B98] Wang X. Sanchez J. Stone M. J. Payne R. J. (2017). Sulfation of the Human Cytomegalovirus Protein UL22A Enhances Binding to the Chemokine RANTES. Angew. Chem. Int. Ed. 56 (29), 8490–8494. 10.1002/anie.201703059 28488292

[B99] Wang Z. A. Cole P. A. (2020). Methods and Applications of Expressed Protein Ligation. Springer US, 1–13. 10.1007/978-1-0716-0434-2_1 PMC7670219 32144660

[B100] Watson E. E. Liu X. Thompson R. E. Ripoll-Rozada J. Wu M. Alwis I. (2018). Mosquito-Derived Anophelin Sulfoproteins Are Potent Antithrombotics. ACS Cent. Sci. 4 (4), 468–476. 10.1021/acscentsci.7b00612 29721529 PMC5920608

[B101] Watson E. E. Ripoll-Rozada J. Lee A. C. Wu M. C. L. Franck C. Pasch T. (2019). Rapid Assembly and Profiling of an Anticoagulant Sulfoprotein Library. Proc. Natl. Acad. Sci. USA 116 (28), 13873–13878. 10.1073/pnas.1905177116 31221752 PMC6628821

[B102] Weeks A. M. Wells J. A. (2020). Subtiligase-Catalyzed Peptide Ligation. Chem. Rev. 120 (6), 3127–3160. 10.1021/acs.chemrev.9b00372 31663725

[B103] Wilkinson B. L. Stone R. S. Capicciotti C. J. Thaysen-Andersen M. Matthews J. M. Packer N. H. (2012). Total Synthesis of Homogeneous Antifreeze Glycopeptides and Glycoproteins. Angew. Chem. Int. Ed. 51 (15), 3606–3610. 10.1002/anie.201108682 22389168

[B104] Yan L. Z. Dawson P. E. (2001). Synthesis of Peptides and Proteins without Cysteine Residues by Native Chemical Ligation Combined with Desulfurization. J. Am. Chem. Soc. 123 (4), 526–533. 10.1021/ja003265m 11456564

[B105] Yang R. Pasunooti K. K. Li F. Liu X.-W. Liu C.-F. (2009). Dual Native Chemical Ligation at Lysine. J. Am. Chem. Soc. 131 (38), 13592–13593. 10.1021/ja905491p 19728708

[B106] Yin H. Zheng M. Chen H. Wang S. Zhou Q. Zhang Q. (2020). Stereoselective and Divergent Construction of β-Thiolated/Selenolated Amino Acids via Photoredox-Catalyzed Asymmetric Giese Reaction. J. Am. Chem. Soc. 142 (33), 14201–14209. 10.1021/jacs.0c04994 32787248 PMC10249021

